# Dual RNA-Seq Unveils *Pseudomonas plecoglossicida htpG* Gene Functions During Host-Pathogen Interactions With *Epinephelus coioides*

**DOI:** 10.3389/fimmu.2019.00984

**Published:** 2019-05-03

**Authors:** Lixing Huang, Lingmin Zhao, Wenjia Liu, Xiaojin Xu, Yongquan Su, Yingxue Qin, Qingpi Yan

**Affiliations:** ^1^Key Laboratory of Healthy Mariculture for the East China Sea, Fisheries College, Jimei University, Ministry of Agriculture, Xiamen, China; ^2^State Key Laboratory of Large Yellow Croaker Breeding, Ningde, China

**Keywords:** pathogen-host molecular integration, dual RNA-seq, *Pseudomonas plecoglossicida*, *Epinephelus coioides*, *htpG*

## Abstract

*Pseudomonas plecoglossicida* is a temperature-dependent opportunistic pathogen which is associated with a variety of diseases in fish. During the development of “white nodules” disease, the expression of *htpG* in *P. plecoglossicida* was found to be significantly up-regulated at its virulent temperature of 18°C. The infection of *htpG*-RNAi strain resulted in the onset time delay, reduction in mortality and infection symptoms in spleen of *Epinephelus coioides*, and affected the bacterial tissue colonization. In order to reveal the effect of *htpG* silencing of *P. plecoglossicida* on the virulence regulation in *P. plecoglossicida* and immune response in *E. coioides*, dual RNA-seq was performed and a pathogen-host integration network was constructed. Our results showed that infection induced the expression of host genes related to immune response, but attenuated the expression of bacterial virulence genes. Novel integration was found between host immune genes and bacterial virulence genes, while *IL6, IL1R2, IL1B*, and *TLR5* played key roles in the network. Further analysis with GeneMANIA indicated that *flgD* and *rplF* might play key roles during the *htpG*-dependent virulence regulation, which was in accordance with the reduced biofilm production, motility and virulence in *htpG*-RNAi strain. Meanwhile, *IL6* and *IL1B* were found to play key roles during the defense against *P. plecoglossicida*, while *CELA2, TRY, CPA1, CPA2*, and *CPB1* were important targets for *P. plecoglossicida* attacking to the host.

## Introduction

To date, infection of ayu (*Plecoglossus altivelis*) (1), large yellow croaker (*Pseudosciaena crocea*) (2), orange spotted grouper (*Epinephelus coioides*) (3), and rainbow trout (*Oncorhynchus mykiss*) (4) with *Pseudomonas plecoglossicida* has been reported. Outbreaks of *P. plecoglossicida* infection in cage-farmed large yellow croaker are mainly recorded in the seawater temperature range of 15–20°C, which is characterized by granulomas in internal organs and lead to severe economic losses (5). In order to find an effective way to prevent or cure the “white nodules” disease, and reduce the economic losses caused by *P. plecoglossicida* infection, it is critical to understand the mechanism underlying *P. plecoglossicida* induced “white nodules” disease (6, 7). Multiple strategies have been adopted to investigate the virulence regulation of *P. plecoglossicida*, including genomics, secretomics, transcriptomics, proteomics, and metabolomics. Moreover, some studies have investigated the immune response of the host to *P. plecoglossicida* infection with RNA-seq. However, these efforts did not directly reflect the intense struggle between the pathogen and the host.

Environmental adaptation has been reported to play an important role in the survival of pathogens, especially in the constantly changing host internal environment (8, 9). When an infection occurs, the gene expression patterns of both the pathogen and the host change dynamically, so that both sides engage in mutual attack (10). Thus, simultaneous monitoring of host-pathogen gene expression profiles during the infection is critical to facilitate a comprehensive understanding of pathogenic mechanisms (11). Hence, the possibility to simultaneously profile host and pathogen transcriptomes via dual RNA-seq in infection biology was proposed in 2012 (12). Recently, dual RNA-seq was successfully used for pathogen-host interactions analysis in *Salmonella typhimurium*-infected HeLa cells (13) and *Streptococcus pneumonia*-infected lung epithelial cells (14). For *S. pneumonia*-lung epithelial cells infection model, dual RNA-seq was used to track transcriptional changes at various time points post-infection, indicating that dynamic transcriptional adjustments are necessary during *in vitro* infection progression (14). However, the study did not examine dynamic interactions between pathogens and the entire host organism during *in vivo* infection progression, which overlooked almost entirely the influence of whole host context on immune response and did not truly reflect the complexities of *in vivo* infection (15). Thus, further studies are required to simultaneously capture the dynamics of transcriptome changes of both a bacterial pathogen and its eukaryotic host during the infection *in vivo* (11). We believe that dual RNA-seq would be an ideal strategy to facilitate our understanding of “white nodules” disease.

Our previous research evaluating the transcriptome, proteome and metabolome of *P. plecoglossicida* incubated under virulent (18°C) and avirulent (28°C) temperatures confirmed that *P. plecoglossicida* was a temperature-dependent facultative pathogen, and showed that *htpG* was significantly highly expressed under 18°C (16, 17). *htpG* encodes the protein HtpG, a member of heat-shock proteins (HSPs) family (18, 19), which was first described in *Escherichia coli* (20). Null mutation of this gene is not lethal to bacterial cells and only results in impaired growth at high temperatures (21). HtpG acts as a chaperone that enables the optimal folding of newly synthesized proteins under stress conditions. The HtpG-mediated folding mechanism does not involve co-chaperones and the identity of proteins acting as substrates in this process remains unknown (22). In *E. coli*, HtpG interacts with the DnaK/DnaJ/GrpE chaperone complex (23). Null mutation of *htpG* affects many physiological processes in *Pseudomonas aeruginosa*, including those that are important for virulence, such as motility, biofilm formation, proteolytic activity, and pigment and biosurfactant production (21). Many virulence factors of *P. aeruginosa* are extracellular and HtpG could play a role in their secretion (21). In addition, HtpG participates in bacterial immunity via the CRISPR system (24), in the virulence of some bacteria like extra-intestinal pathogenic *E. coli* strains, *Edwardsiella tarda*, or *Salmonella typhimurium*, and plays a role in the biosynthesis of antibiotic and toxins (25–27). The role of *htpG* remains highly elusive in bacterial pathogenicity, and its function during pathogen infection has not been reported (28).

Given the major harm caused by *P. plecoglossicida* to cultured fish and the potential important role of *htpG* in its virulence, the virulence of wild-type and *htpG*-RNAi *P. plecoglossicida* to *E. coioides* were compared. The spleens of *E. coioides* infected with wild-type and *htpG*-RNAi *P. plecoglossicida* were subjected to dual RNA-seq for further comparison. The aim of this study was to reveal the effect of *htpG* silencing of *P. plecoglossicida* on the virulence regulation in *P. plecoglossicida* and immune response in *E. coioides*. To the best of our knowledge, this was the first attempt to use tissue dual RNA-seq to simultaneously monitor the dynamics of gene expression changes of a bacterial pathogen and its eukaryotic host and infer molecular inter-species interactions between them, which could simultaneously obtain high-resolution dynamic transcriptome data from the interacting species in prokaryotic and eukaryotic biological systems, as well as predict molecular inter-species interactions based on the dual-transcriptomics data, and further predict and identify crucial virulence genes during infection.

## Materials and Methods

### Bacterial Strains and Culture Conditions

The pathogenic *P. plecoglossicida* strain (NZBD9) was isolated from the spleen of naturally infected large yellow croaker (2). Physiological saline with 10% glycerol was used to store the NZBD9 strain at −80°C. The NZBD9 strain was routinely grown in Luria Bertani (LB) medium at 18 or 28°C with shaking at 220 rpm. *E. coli* DH5α was obtained from TransGen Biotech (Beijing, China), and grown in LB medium (37°C, 220 rpm).

### Construction of *P. plecoglossicida* RNAi Strain

RNAi strain was constructed according to previously described methods (5). Five short hairpin RNA sequences targeting *htpG, rplF*, and *flgD* were designed and synthesized by Shanghai Generay Biotech Co., Ltd. (Shanghai, China) (Table S1). After linearizing pCM130/tac vectors with the restriction enzymes *Nsi*I and *Bsr*GI (New England Biolabs, U.S.A), the oligonucleotides were annealed and ligated to the linearized pCM130/tac vectors using T4 DNA ligase (New England Biolabs) based on the manufacturer's recommendations. The recombinant pCM130/tac vectors were transformed into competent *E. coli* DH5a cells by heat shock, and then extracted and electroporated into *P. plecoglossicida*. Finally, the expression level of the target gene of each RNAi strain was evaluated by qRT-PCR.

### Artificial Infection and Sampling

*E. coioides* (average weight 50.0 ± 2.0 g) fish were obtained from Zhangzhou (Fujian, China) and acclimatized at 18°C for 1 week under specific pathogen-free laboratory conditions. Fish were tested to be healthy by sera agglutination and bacteriological recovery tests as described by Pang et al. (29). *E. coioides* were divided into several groups (n = 40 in each group, triplicates were used for each experiment) and grown in 500 L tanks with constant aeration and a flow-through set-up.

For survival assays, weight-matched *E. coioides* were intrapleurally injected with 10^3^ cfu/g of *P. plecoglossicida* (wild-type or RNAi strain). *E. coioides* that were intrapleurally injected with PBS were used as negative control. The water temperature during infection was 18 ± 1°C. The daily mortality of infected *E. coioides* was recorded. For dual RNA-Seq, the spleens of six weight-matched *E. coioides* infected with wild-type *P. plecoglossicida* or RNAi strain were sampled at 48 h post-infection (hpi). Every two spleens were mixed as one sample. For the tissue distribution assays, the spleens, livers, head kidneys, trunk kidneys and blood of three *E. coioides* were sampled at 24, 48, 72, and 96 hpi, respectively.

### DNA Isolation

DNA from spleens, livers, head kidneys and trunk kidneys was purified with an EasyPure Marine Animal Genomic DNA kit (TransGen Biotech, Beijing, China) according to the manufacturer's instructions (30). The EasyPure Blood Genomic DNA kit (TransGen Biotech) was used for DNA isolation from blood samples.

### RNA Isolation

Total RNA was extracted using TRIzol reagent (Invitrogen, Carlsbad, CA, USA) according to the manufacturer's instructions. The mixed genomic DNA in total RNA was digested with the Turbo DNA-free DNase (Ambion, Austin, TX, USA) (31). The RNA quality was assessed using an Agilent 2100 Bioanalyzer (Agilent Technologies, Santa Clara, CA, USA). The rRNA in total RNA was removed using the Ribo-Zero rRNA removal kit (Epicenter, Madison, WI, USA) according to the manufacturer's instructions (32).

### Quantitative Real-Time PCR (qRT-PCR)

qRT-PCR was performed using a QuantStudio 6 Flex real-time PCR system (Life Technologies, USA) (33). All primer sequences are listed in Table S2. The expression of bacterial genes was normalized using *16s* rDNA. In *E. coioides*, the expression of genes was normalized to β*-actin*. The 2^−ΔΔ*Ct*^ method was used to calculate the relative levels of gene expression (34).

To evaluate the dynamic distribution of *P. plecoglossicida* in host, the copy number of the *gyrB* gene was used to estimate *P. plecoglossicida* abundance in spleens, trunk kidneys, head kidneys, blood and livers. The primers are listed in Table S2. The *gyrB* DNA copy number per milligram of tissue was used to express the results.

### Transcriptomic Analysis

#### Library Preparation and Sequencing

The RNA-seq libraries were prepared using protocols supplied with the TruSeq™ RNA sample preparation kit (Illumina, San Diego, CA, USA). Briefly, the rRNA-depleted RNA sample was fragmented in fragmentation buffer, and cDNA was synthesized using a SuperScript double-stranded cDNA synthesis kit (Invitrogen, Carlsbad, CA, USA). After end reparation, phosphorylation and poly (A) addition, the cDNA library was amplified using Phusion DNA polymerase (New England Biolabs). An Agilent 2100 Bioanalyzer (Agilent Technologies) was used to validate the library quality. Sequencing was performed on the Illumina HiSeq4000 sequencing platform at Majorbio Biotech Co., Ltd. (Shanghai, China).

#### Processing and Mapping of Reads

The trimming and quality control of raw Illumina reads were performed using Sickle (https://github.com/najoshi/sickle) and SeqPrep (https://github.com/jstjohn/SeqPrep), with the default settings. For RNA-seq, clean data were mapped to the genome of *P. plecoglossicida* strain NZBD9 [NCBI Sequence Read Archive (SRA) under accession number SRP062985] using Bowtie2 (35). Mapped reads were classified as reads of *P. plecoglossicida*, and leftover reads were used for *de novo* assembly to obtain the *E. coioides* unigenes.

#### *De novo* Assembly and Annotation of mRNAs in the Host

All clean reads, which were not mapped to the *P. plecoglossicida* genome from the wild-type and RNAi strain-infected spleens, were treated as a pool of reads. This pool was assembled *de novo* into unigenes using Trinity (36). To remove any possible prokaryote contamination, all unigenes were first aligned to the bacterial NCBI non-redundant (NR) protein database. For annotation of mRNAs, the clean unigenes were compared with different databases, including NCBI NR protein, STRING, SWISS-PROT and Kyoto Encyclopedia of Genes and Genomes (KEGG) using BLASTX to identify the proteins that shared the highest sequence similarity with the identified unigenes. Gene Ontology (GO) annotations were conducted using the Blast2GO software (http://www.blast2go.com) (37). Finally, KEGG was used for metabolic pathway analysis (http://www.genome.jp/kegg/) (38).

#### Analysis of Differential Gene Expression

Expression analyses of transcriptome data from *E. coioides* were performed based on the annotations from NCBI (NZ_ASJX00000000.1) and the reference transcriptome annotation described above (annotation for the host transcriptome), respectively. After obtaining uniquely mapped read counts, the package edgeR (targeting mRNAs of host) (39) was used to test for differentially expressed genes (DEGs), which were determined by the following thresholds: |*log*2^*foldchange*^| ≥ 1 and a false discovery rate (FDR) <0.05.

#### Analysis of *htpG*-Dependent Virulence Gene Distribution and Their Transcription Factors

To classify the gene distribution of the *htpG*-dependent proteins derived from the dual RNA-seq, we adopted the following filtration analysis (40). First, a gene cluster is defined as at least two neighboring genes and tolerant to only one gene missed among at least three genes in a cluster. Second, the genes must be located on the same DNA sequence strand, either positive or negative. Finally, the distance between the neighboring members of a gene cluster must be within −50 to +100 base pairs.

When the clusters were identified, we identified the starting position of the first gene in each gene cluster and submitted all the sequences on the scaffolds corresponding to that location for transcription factor prediction by BPROM (http://linux1.softberry.com/berry.phtml?topic=bprom&group=programs&subgroup=gfindb).

#### Prediction of a Gene Regulatory Network in Pathogen-Host Interactions

Network inference was implemented using the NetGenerator algorithm (41, 42). Briefly, the DEGs from the pathogenic enriched pathways of modules in *P. plecoglossicida* and the immune response-related pathways in *E. coioides* were used for the prediction of a gene regulatory network. The robustness of the putative gene regulatory network was checked, and edges identified by >50% of the iterations were chosen as the final network.

#### Gene Co-expression Network Prediction

For candidate modules, a gene co-expression network was constructed, where a gene was represented using each node and co-expression correlation was expressed as the connecting line (edge) between two genes. The nodes of most connections in the gene network were considered as hub genes. The Cytoscape software was used to visualize the network.

### Growth Curves

The wild-type and gene-silenced *P. plecoglossicida* were grown at 28°C in LB overnight and then the OD_600nm_ of the cultures was adjusted to 0.2. The *P. plecoglossicida* strains were then incubated at 18°C (*n* = 3). The values of OD_600nm_ were measured at 0, 3, 6, 9, 12, 15, 18, 21, 24 h, and growth curves were plotted to compare the wild-type and silenced strains (43).

### Biofilm Assay

The biofilm assay for *P. plecoglossicida* was conducted as previously described (44). *P. plecoglossicida* strains were grown at 28°C in LB overnight and then the OD_600nm_ of the cultures was adjusted to 0.2.150 μl of LB was mixed with 50 μl of bacterial culture, and then incubated at 18°C. After incubation for 24 h, it was washed three times using sterile PBS, stained for 15 min with 200 μl 1% crystal violet, rinsed again with sterile PBS, and then air dried. Two hundred microliter acetic acid (33%) was used for solubilizing the stained biofilm, which was quantitated by measuring OD_590nm_. Six replicates were performed for each treatment.

### Soft Agar Plate Motility Assay

To assay the motility of *P. plecoglossicida* strains, the soft agar method was adapted (45). Overnight cultures were diluted to OD_660_ = 0.03. 1 μl drop of the cell suspension was spotted on to the center of the LB plates (0.3% agar) at 37°C for 20 h. The diameters of the colonies were measured at 24 h.

### Statistical Analyses

All data are expressed as the means ± standard deviation (SD) from at least three sets of independent experiments. Data analysis was performed using the SPSS 17.0 software (Chicago, IL, USA), and one-way analysis of variance with Dunnett's test were used. *P* < 0.05 was considered statistically significant.

Associations between the expression of hub virulence genes and *htpG* in the spleen during the infection were analyzed with bivariate correlations tests followed by Pearson's correlation coefficient via SPSS 18.0 software. *P* < 0.05 was used to indicate significant correlation.

### Data Access

The RNA sequencing reads data were deposited at the GenBank SRA database under the accession number PRJNA497501.

### Ethics Statement

All animal experiments were conducted strictly based on the recommendations in the “Guide for the Care and Use of Laboratory Animals” set by the National Institutes of Health. The animal protocols were approved by the Animal Ethics Committee of Jimei University (Acceptance No. JMULAC201159).

## Results

### Construction of the *htpG*-RNAi Strain

**Figure 1:**
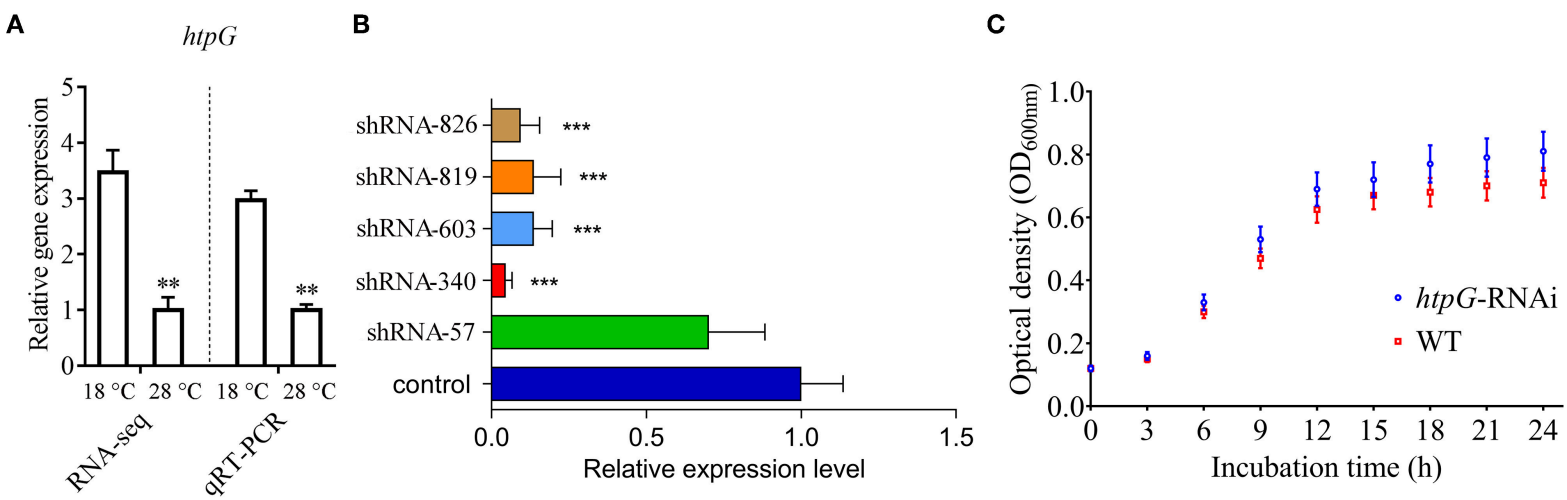
Construction and growth curve of *htpG*-RNAi strain of *P. plecoglossicida*. **(A)** Expression of *htpG* at 18 and 28°C in wild-type strain. **(B)** The *htpG* expression levels of 5 *htpG*-RNAi silencing strain. **(C)** Growth curve of wild-type strain and *htpG*-RNAi strain. Data are shown as means ± SD from three independent biological replicates. **P < 0.01, ***P < 0.001.

qRT-PCR results showed that the expression of *P. plecoglossicida htpG* was significantly higher at 18°C than at 28°C *in vitro* (Figure 1A), which was consistent with the results of previous RNA-seq.

Four of the five shRNAs significantly reduced the expression of *htpG*, with different efficiencies (Figure 1B). The *htpG*-RNAi-340 strain exhibited the best efficiency of gene silencing, and was chosen for further studies. Although *htpG* was silenced, *htpG*-RNAi strain growth rate was similar to the control strain (Figure 1C).

### Effect of *htpG* Gene on the Virulence of *P. plecoglossicida*

**Figure 2:**
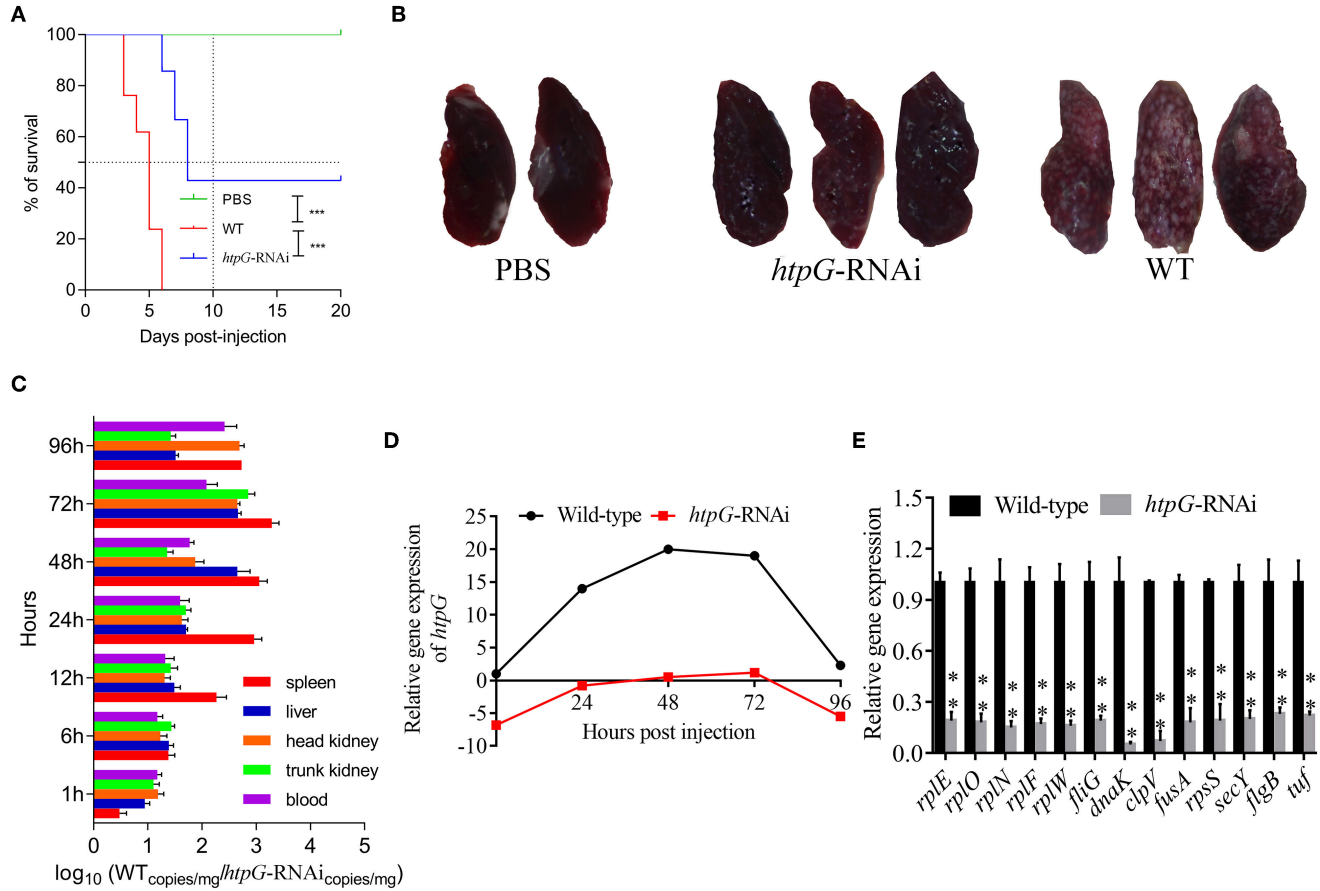
The virulence of wild-type and *htpG*-RNAi strain of *P. plecoglossicida*. **(A)** Survival rate of *E. coioides* infected by *P. plecoglossicida* (*n* = 3). **(B)** Symptoms of spleen of *E. coioides* infected by *P. plecoglossicida*. **(C)** Spatial and temporal distribution of *htpG*-RNAi strain compared to wild-type strain. **(D)** Expression level of *htpG* of *P. plecoglossicida* in the spleen of *E. coioides*. **(E)** qRT-PCR analysis of the expression of randomly selected novel genes among wild type *P. plecoglossicida in vitro*, wild type *P. plecoglossicida in vivo*, and *htpG-RNAi* strain *in vivo*. Data are presented as mean ± S.D. (*n* = 3). **P < 0.01, ***p < 0.0001.

As compared to the counterparts injected with wild-type *P. plecoglossicida*, the *E. coioides* injected with *htpG*-RNAi strain exhibited a significant delay in the time of death and a significant decrease in mortality (Figure 2A). At 96 hpi, the spleens of the *E. coioides* injected with wild-type *P. plecoglossicida* showed typical symptoms (the surface of the spleen was covered with numerous white spots), while white spots were almost undetectable on the surface of spleens of *E. coioides* injected with RNAi strain (Figure 2B).

The dynamic distribution of *htpG*-RNAi strain and wild-type strain of *P. plecoglossicida* in *E. coioides* was compared (Figure 2C). The abundances of *htpG*-RNAi strain in the spleen, liver, head kidney, trunk kidney, and blood were significantly reduced as compared to the wild-type *P. plecoglossicida* at majority of the time points post-injection.

The expression level of *htpG* of wild-type and *htpG*-RNAi strains in spleens at different times post-injection under 18°C was evaluated (Figure 2D, Figure S1). The expression level of *htpG* gene in the spleen infected with wild-type strain peaked at 48 hpi and then gradually decreased (Figure S1). For 96 h post-infection, the expression of *htpG* in *htpG*-RNAi strain was always lower than wild-type strain in the spleen (Figure 2D).

### RNA-Seq of *P. plecoglossicida* in Infected Spleen of *E. coioides*

The gene expression profile was calculated by edgeR, and there was obvious statistical difference when the changes in the expression level that met FDR <0.05 & |log2FC|≥1. According to the heat map generation and hierarchical clustering, the individual strain repeats of *P. plecoglossicida*, naturally separated and clustered according to their respective sample groups, which indicated the remarkable transcriptomic alterations among wild-type *P. plecoglossicida in vitro* and *in vivo*, and *htpG-RNAi* strain *in vivo*. As compared to wild-type *P. plecoglossicida in vitro* and *in vivo*, numerous genes were down-regulated in *htpG-RNAi* strain *in vivo*, which indicated that the *htpG* gene probably plays an important role in the pathogenesis of *P. plecoglossicida* by regulating other genes. A total of 4,914 genes were identified from profiled transcripts of *P. plecoglossicida* in infected spleen of *E. coioides*. As compared to the wild-type *P. plecoglossicida* in infected spleen of *E. coioides*, 159 genes from the *htpG-RNAi* strain in infected spleen of *E. coioides* were significantly differentially expressed, with 157 down-regulated genes and two up-regulated genes (Figure S2).

To validate the results from the transcriptomic analysis, qRT-PCR was performed. As compared to the wild-type strain *in vivo*, the qRT-PCR results revealed significant down-regulation of *rplE, rplO, rplN, rplF, rplW, fliG, dnaK, clpV, fusA, rpsS, secY, flgB*, and *tuf* in *htpG-RNAi* strain *in vivo* (Figure 2E). The qRT-PCR results matched those of the sequencing, which reinforced the reliability of the RNA-seq.

The functions of these 159 differentially expressed genes (DEGs) were analyzed by GO, and the number of genes mapped to every term was counted. This analysis categorized the 159 DEGs into 30 enriched functional groups. Most of the DEGs were involved in the following functional categories: cellular process, metabolic process, single-organism process, cell, cell part, macromolecular complex, organelle, binding, and catalytic activity.

Pathway analysis of DEGs based on the latest version of the KEGG database was also conducted. According to the KEGG database, the 159 DEGs were enriched in 36 KEGG pathways, including pathways closely related to bacterial virulence regulation such as flagellar assembly, RNA degradation, ribosome, TCA cycle, glycolysis, and plant-pathogen interaction.

**Figure 3:**
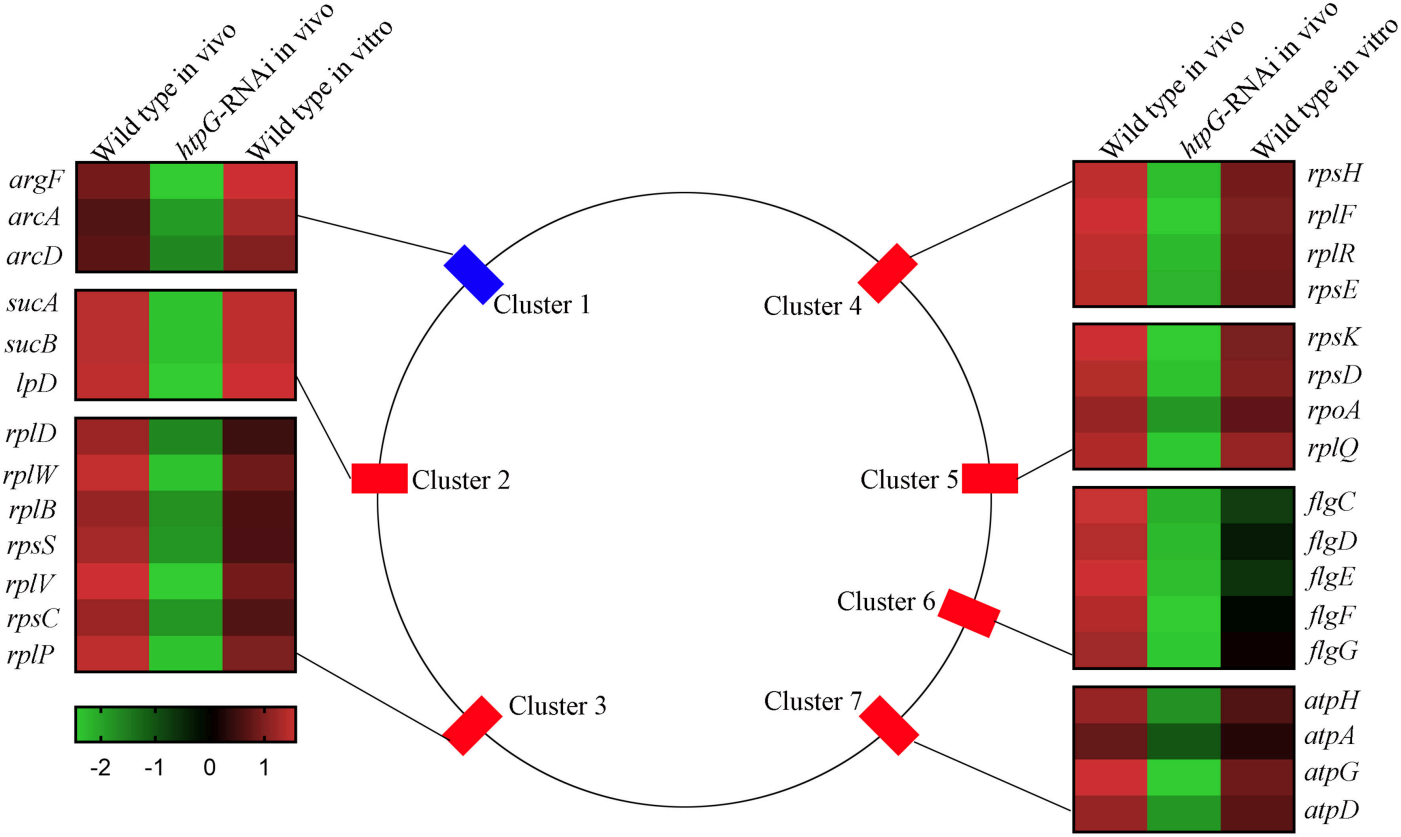
The genomic localization for the *P. plecoglossicida* DEGs included in group 1 that were filtered by cluster analysis. Central panel represents the genomics localization for the DEGs. Right and left panels represent the fold changes of abundance of each DEG. The lower panel with color gradient represents the changes of gene abundance from down-regulated (green) to up-regulated (red).

To elucidate *htpG*-related gene expression profiles, the 159 DEGs were further analyzed by K-Means Cluster analysis. Generally, K-means cluster analysis is based on the assumption that genes involved in either a similar function or a common pathway will have similar expression profiles and can likely be grouped together. The analysis was performed among wild-type *P. plecoglossicida in vitro* and *in vivo*, and *htpG-RNAi* strain *in vivo*. The 159 DEGs were clustered into five primary clusters and then grouped into three different groups based on significant changes in expression patterns (Figure S3). Group 1, the largest group, consisted of 155 genes that were further divided into two sub-groups. All the genes in this group exhibited low expression levels in *htpG-RNAi* strain *in vivo*, but high levels in wild-type *P. plecoglossicida in vitro* and *in vivo*. Two DEGs from group 2 showed similar expression pattern as group 1, but were slightly down-regulated in wild-type *P. plecoglossicida in vitro*. Group 3 included two DEGs, which showed elevated expression in *htpG-RNAi* strain *in vivo* and low expression in wild-type *P. plecoglossicida in vitro* and *in vivo*. According to the results of K-Means Cluster analysis, DEGs included in group 1 seemed to be positively related to *htpG*, whose distribution was classified into seven gene clusters covering 30 genes (Figure 3).

Cluster 1 consisted of three genes, including *argF, arcA*, and *arcD*, which belonged to the arginine biosynthesis pathway. Cluster 2 consisted of three genes, including *sucA, sucB*, and *lpD*, which belonged to the TCA cycle pathway. Cluster 3 (including *rplD, rplW, rplB, rpsS, rplV, rpsC*, and *rplP*), Cluster 4 (including *rpsH, rplF, rplR*, and *rpsE*) and Cluster 5 (including *rpsK, rpsD, rpoA*, and *rplQ*) consisted of seven genes, four genes and four genes, respectively, which encoded the ribosomal proteins. Cluster 6 consisted of five flagellar assembly genes, including *flgC, flgD, flgE, flgF*, and *flgG*. Cluster 7 consisted of four genes, including *atpH, atpA, atpG*, and *atpD*, which belonged to the oxidative phosphorylation pathway.

Because of genes involved in ribosome assembly and flagellar assembly were usually chief virulence genes, the 20 genes belonging to Clusters 3, 4, 5, and 6 were chosen for further analysis of pathogen-host interactions.

### Tissue RNA-Seq of Infected Spleen of *E.coioides*

There was obvious statistical difference when the changes in the expression level that met FDR <0.05 & |log2FC|≥1. According to the heat map generation and hierarchical clustering, the individual samples of *E. coioides* spleen, naturally separate and cluster according to their respective sample groups, which indicated the remarkable transcriptomic alterations among *E. coioides* injected with PBS, wild-type *P. plecoglossicida* and *htpG-RNAi* strain. This indicated that under these different conditions, the physiological status of the host was very different, and these differences might be closely related to the immune response of the host. A total of 180,502 genes were identified from profiled transcripts of *E. coioides* spleen. As compared to the wild-type *P. plecoglossicida* infected spleen of *E. coioides*, 17,512 genes from the *htpG-RNAi* strain infected spleen of *E. coioides* were significantly differentially expressed, with 10,694 down-regulated genes and 6,818 up-regulated genes (Figure S4). The large number of DEGs indicated that *htpG* gene plays an important role in the immune response of *E. coioides* to *P. plecoglossicida*.

**Figure 4:**
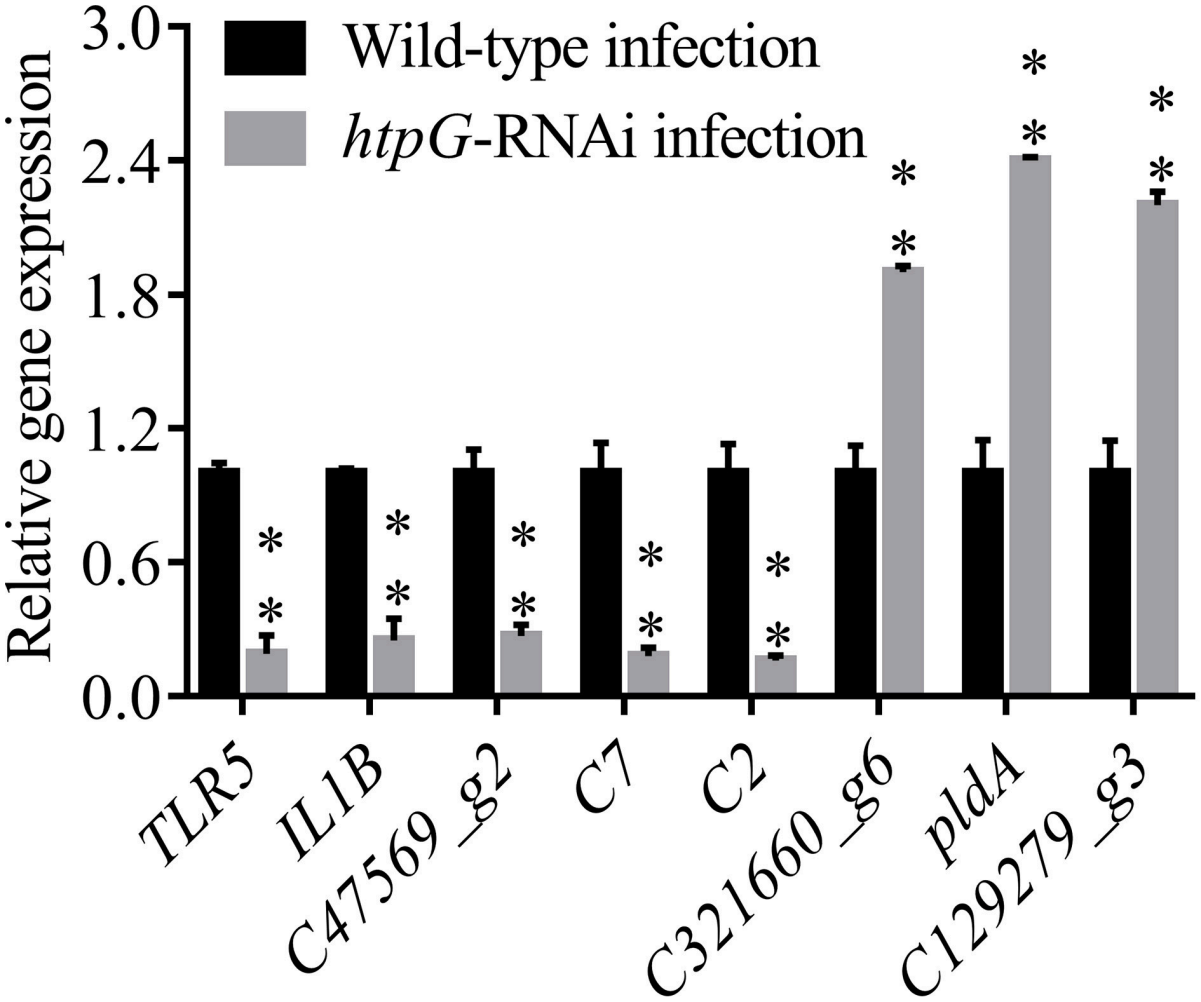
qRT-PCR analysis of the expression of randomly selected novel genes among *E. coioides* accepted injection of PBS, wild type *P. plecoglossicida* and *htpG-RNAi* strain. Data are presented as mean ± S.D. (*n* = 3). **P < 0.01.

To validate the results from the transcriptomic analysis, qRT-PCR was performed. As compared to the wild-type *P. plecoglossicida* infected spleen of *E. coioides*, the qRT-PCR results revealed significant down-regulation of *TLR5, IL1B, C47569_g2, C7* and *C2*; and significant up-regulation of *C321660_g6, pldA* and *C129279_g3* in *htpG-RNAi* strain infected spleen of *E. coioides* (Figure 4). The qRT-PCR results matched the sequencing results, which reinforced the reliability of the RNA-seq.

The functions of the 17,512 DEGs were analyzed by GO, and the number of genes mapped to every term was counted. This analysis categorized the 17,512 DEGs into 56 enriched functional groups while some DEGs were involved in the immune response such as cell killing, immune system, and response to stimulus.

Pathway analysis of DEGs based on the latest version of the KEGG database was also conducted. According to the KEGG database, the 17,512 DEGs were enriched in 67 KEGG pathways, including pathways closely related to Immunoregulation such as cytokine-cytokine receptor interaction, complement and coagulation cascades, lysosome, intestinal immune network for IgA production, systemic lupus erythematosus, primary immunodeficiency, NF-kappa B signaling pathway, antigen processing and presentation, chemokine signaling pathway, and Toll-like receptor signaling pathway.

K-Means Cluster analysis was performed on the 17,512 DEGs among *E. coioides* injected with PBS, wild-type *P. plecoglossicida* and *htpG-RNAi* strain. The 17,512 DEGs were clustered into 10 primary clusters and then grouped into five different groups based on significant changes in expression patterns (Figure S5). Group 1, the largest group, consisted of 17,319 genes, which displayed horizontal expression profiles among samples. Group 2 consisted of 58 genes, which exhibited high expression levels in healthy *E. coioides*, but low levels in *E. coioides* injected with wild-type *P. plecoglossicida* and *htpG-RNAi* strain. Meanwhile, genes in this group exhibited slightly elevated expression in *E. coioides* injected with *htpG-RNAi* strain than those injected with wild-type *P. plecoglossicida*. This indicated that *P. plecoglossicida* probably attenuated the expression of these genes in the host, which might be one of the key pathogenic mechanisms of *P. plecoglossicida* regulated by *htpG*. Moreover, *htpG* was critical for this modulation. KEGG analysis showed that genes in this group were involved in the metabolism of proteins, fats and other substances in the host. Group 3 consisted of 82 genes, which exhibited low expression in healthy *E. coioides*, but high expression in *E. coioides* injected with wild-type *P. plecoglossicida* and *htpG-RNAi* strain. This indicated that the expression of *P. plecoglossicida htpG* gene had no significant effect on the expression of genes involved in Group 3, but this part of the host gene must be involved in the host's defense against *P. plecoglossicida* infection, which is the key to the host's resistance to *P. plecoglossicida*. Subsequent studies with KEGG database showed that these genes were involved in immune defense-related processes such as host complement production, interleukin production and signaling, and gene expression regulation. Group 4 consisted of 29 genes, whose expression levels in *E. coioides* injected with wild-type *P. plecoglossicida, htpG-RNAi* strain and PBS decreased in turn. This indicated that the expression of *htpG* activated the expression of these host genes. Therefore, this part of the gene may be another key to pathogenesis of *P. plecoglossicida* regulated by *htpG*, or it may be involved in interactions between *P. plecoglossicida* and host immune system regulated by *htpG*. KEGG analysis revealed that these genes were involved in immune defense-related processes including cytokine-cytokine receptor interaction, intestinal immune network for IgA production, and Toll-like receptor signaling pathway. Group 5 consisted of 24 genes that were further divided into six sub-groups. Genes in this group did not show obvious regularity. Based on these findings, the protein metabolism-related genes from Group 2, and immune response-related genes from Groups 3 and 4, were chosen for further analysis of pathogen-host interactions.

### Prediction of *htpG*-Dependent Key Genes in Pathogen-Host Interactions

To predict the *htpG*-dependent key genes of *P. plecoglossicida* involved in infection, the genes from *P. plecoglossicida* and *E. coioides* mentioned above were subjected to further analysis.

**Figure 5:**
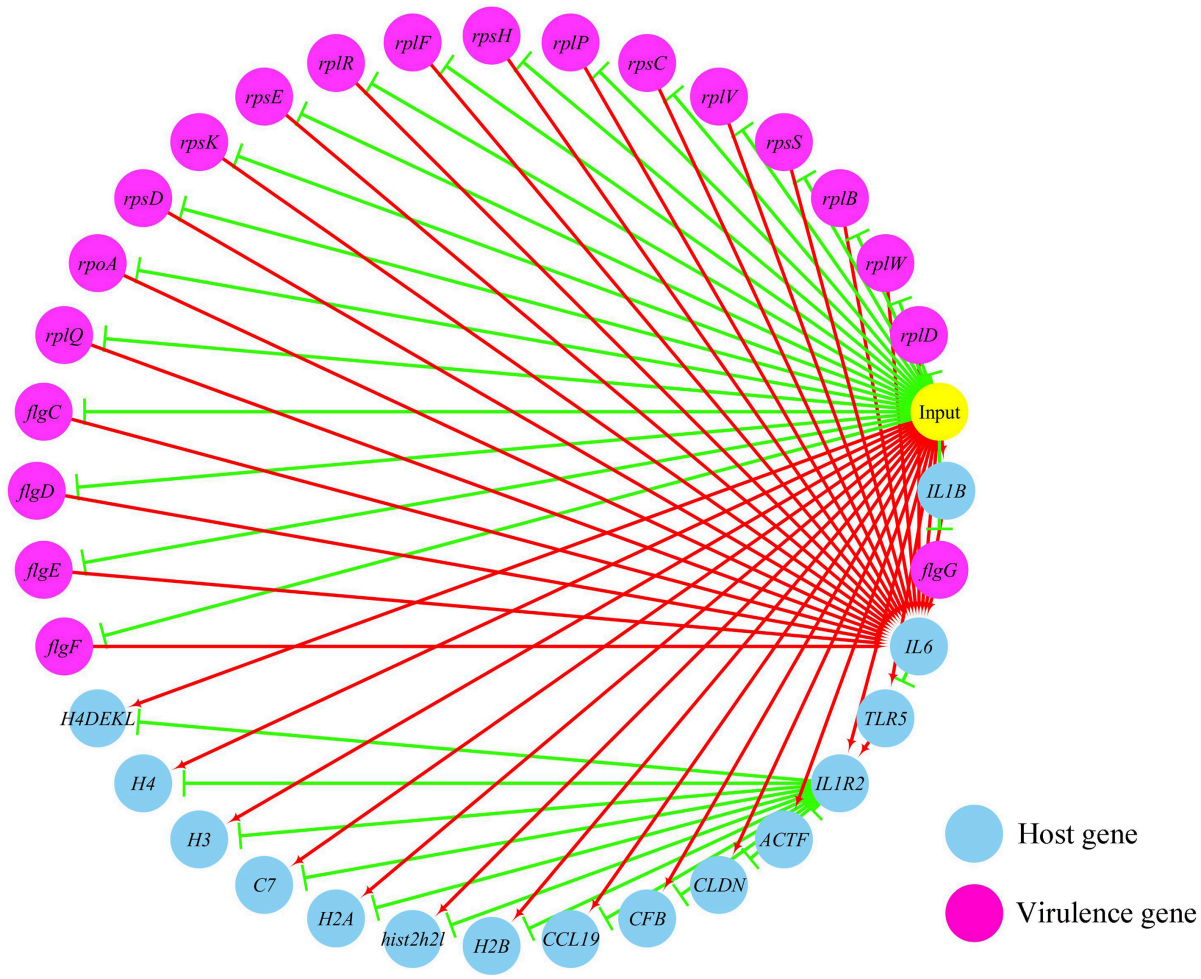
The predicted regulation network between the virulence genes and host immune response related genes. For the predicted regulation network, the yellow circle represents the occur of infection; each blue circle represents an input host immune response related genes; each purple circle represents an input virulence gene; each red line represents a positive correlation; and each green line represents a negative correlation.

**Figure 6:**
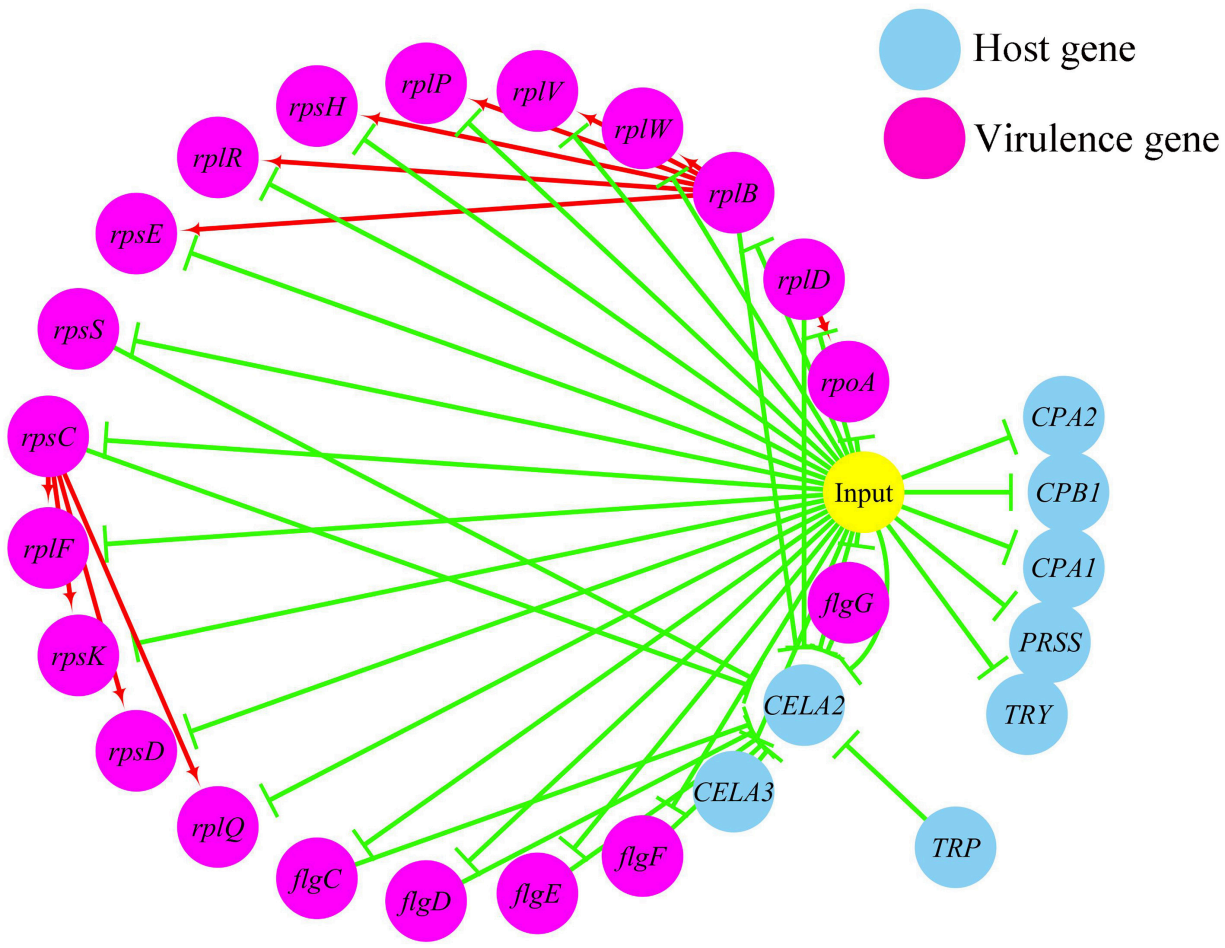
The predicted regulation network between the virulence genes and host protein metabolism related genes. For the predicted regulation network, the yellow circle represents the occur of infection; each blue circle represents an input host immune response related genes; each purple circle represents an input virulence gene; each red line represents a positive correlation; and each green line represents a negative correlation.

**Figure 7:**
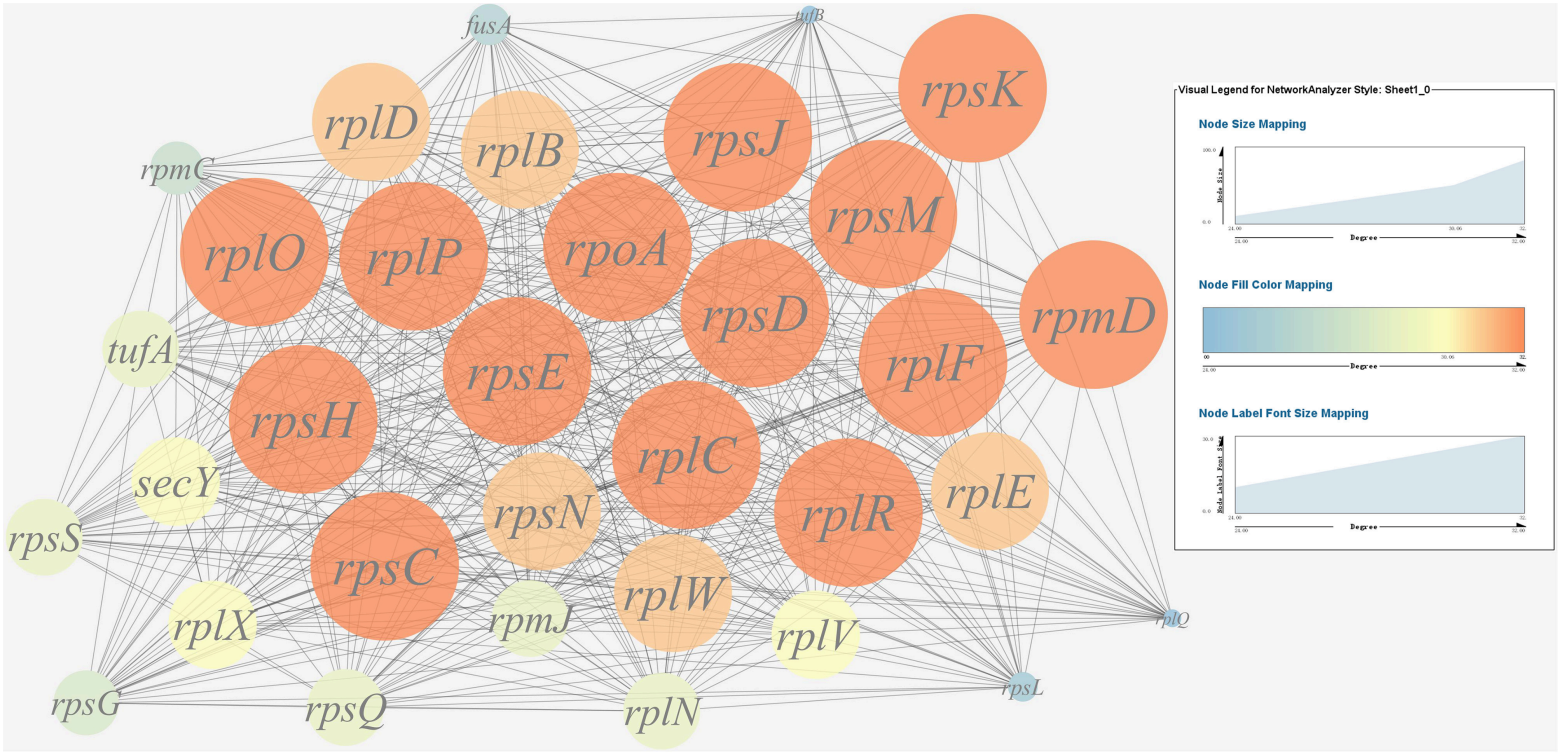
Gene co-expression network of ribosomal genes in *P. plecoglossicida*. For the predicted regulation network, each circle represents a gene; each line represents a correlation. For the genes, the diameter, color and symbol size of each node is directly proportional to the number of nodes associated with it. Among ribosomal genes, *rpsM, rpsJ, rplC, rplO, rpmD, rpsD, rpsE, rpoA, rpsC, rplR, rpsH, rplP, rpsK*, and *rplF* were the nodes of most connections in the network and considered as hub genes.

**Figure 8:**
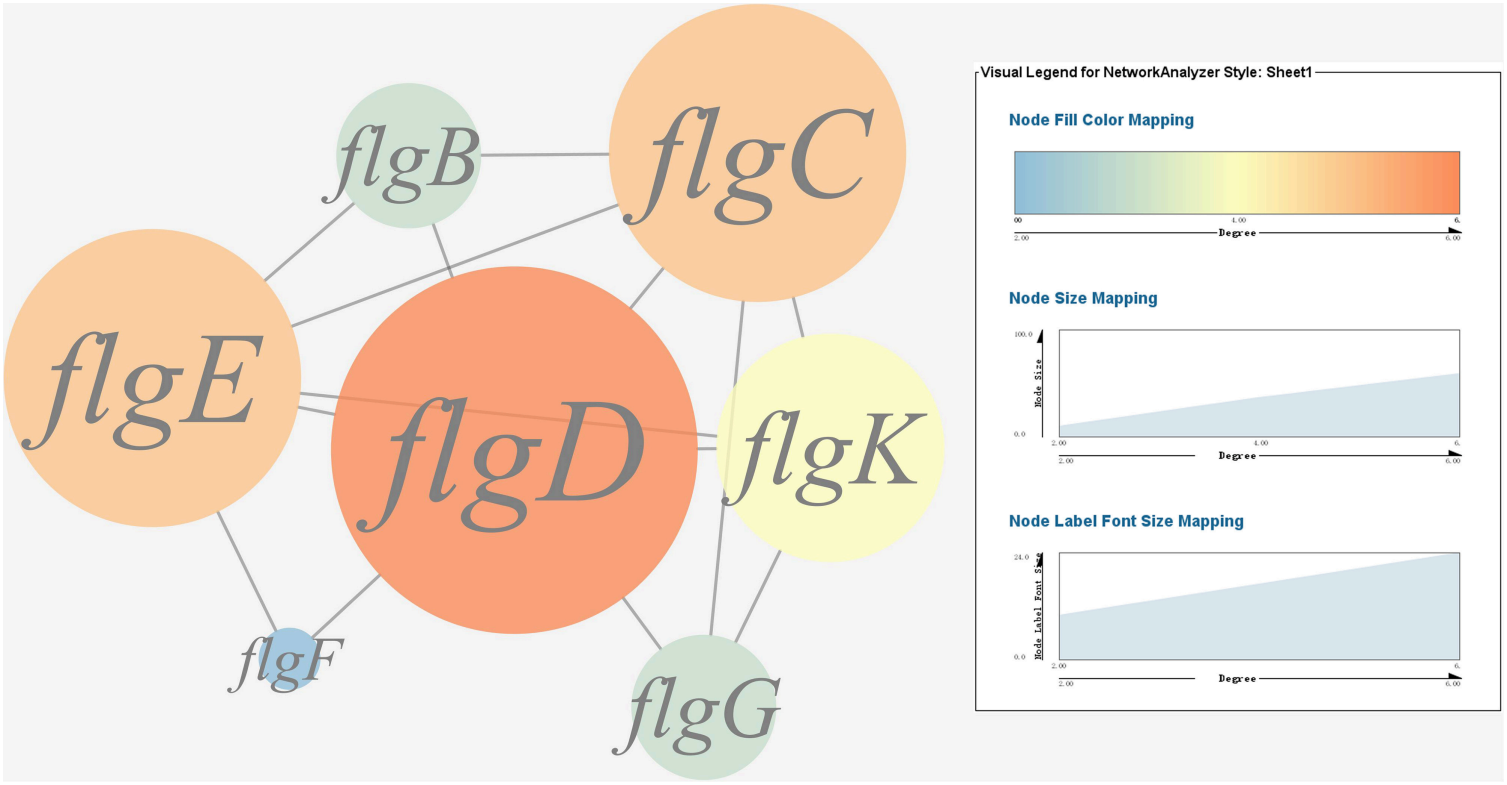
Gene co-expression network of flagellar genes in *P. plecoglossicida*. For the predicted regulation network, each circle represents a gene; each line represents a correlation. The candidate key virulence gene *flgD* is marked in red.

**Figure 9:**
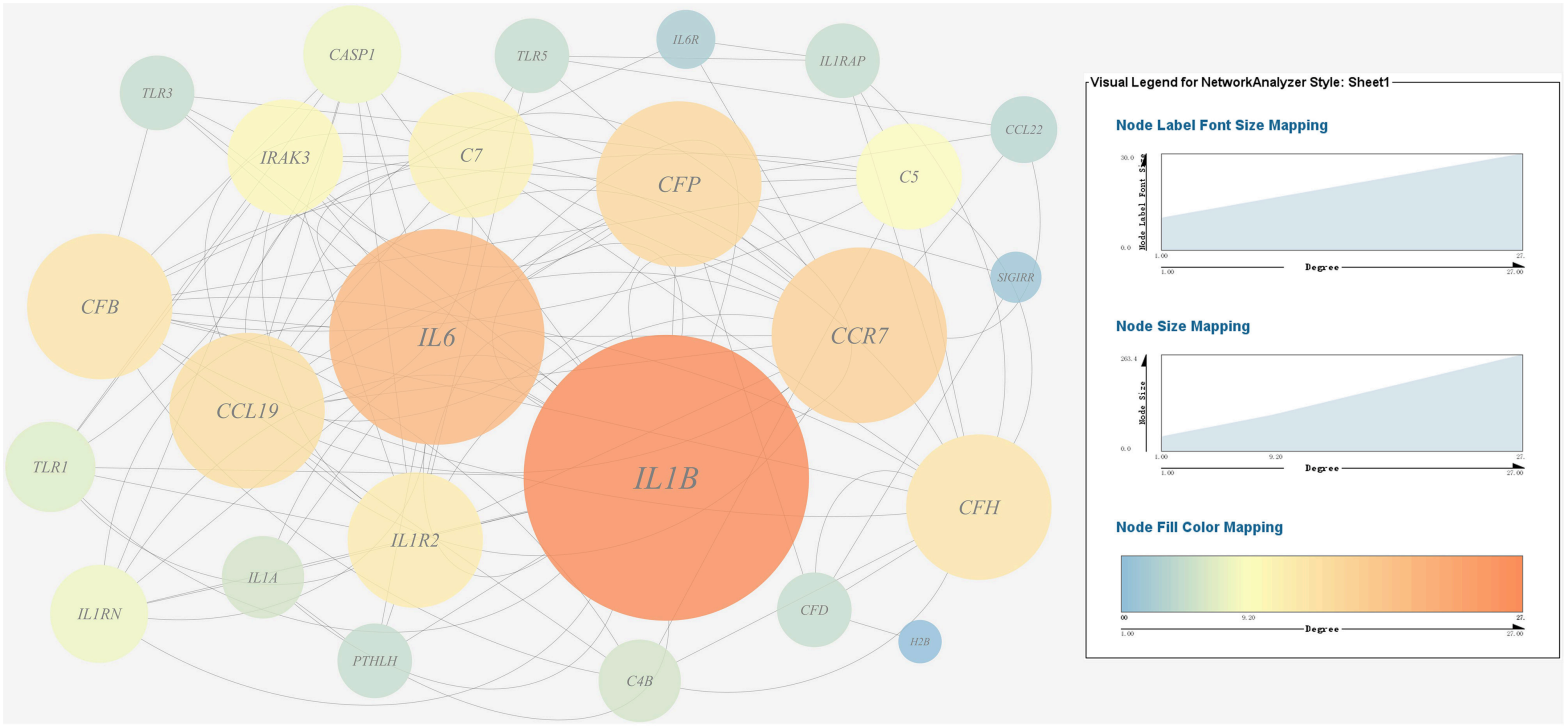
Gene co-expression network of host immune response related genes. For the predicted regulation network, each circle represents a gene; each line represents a correlation. For the genes, the diameter, color and symbol size of each node is directly proportional to the number of nodes associated with it. Among these genes, *IL6* and *IL1B* were the nodes of most connections in the network and considered as hub genes.

**Figure 10:**
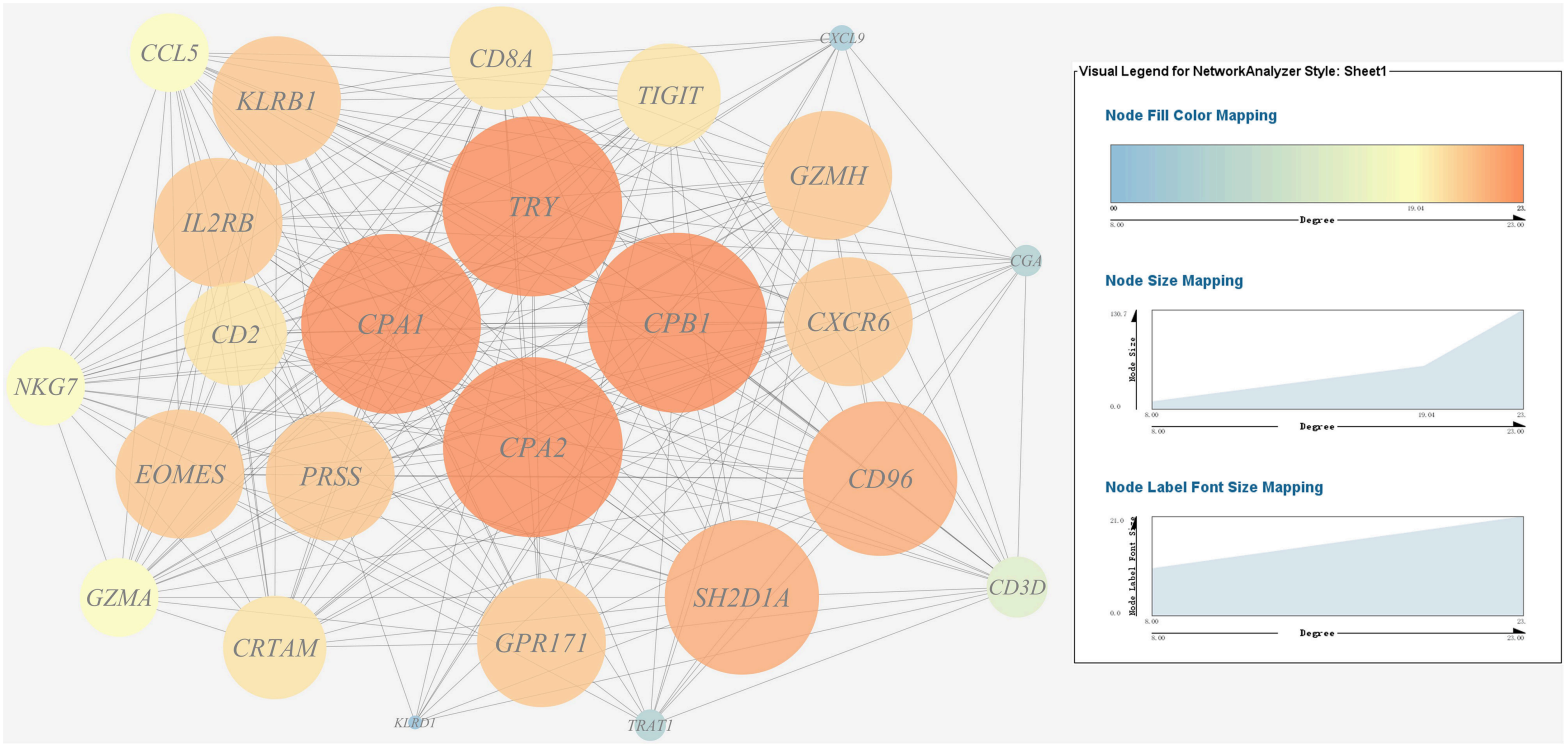
Gene co-expression network of host protein metabolism related genes. For the predicted regulation network, each circle represents a gene; each line represents a correlation. For the genes, the diameter, color and symbol size of each node is directly proportional to the number of nodes associated with it. Among these genes, *TRY, CPA1, CPA2*, and *CPB1* were the nodes of most connections in the network and considered as hub genes.

(1) Prediction of gene regulatory networks in pathogen-host interactions.
  Figures 5, 6 show the pathogen-host gene regulatory prediction networks. In terms of the correlations between the virulence genes and host immune response-related genes (Figure 5), first of all, occurrence of infection activated the expression of immune-related genes, which then attenuated the expression of virulence genes. Secondly, there were interactions between pathogenic genes and host genes. Virulence genes such as *rplD, rplW, rplQ, flgD*, and *flgE* could influence host immunity and thus activate the expression of *IL6*, indicating that *IL6* played a key role in the interaction between pathogen and host. Finally, there were mutual regulatory relationships between host genes, such as *IL6* inhibited *TLR5*; *TLR5* promoted *IL1R2*; *IL1R2* inhibited *H4DEKL, H4, H3, C7, H2A, H2B, CCL19, CFB, CLDN*, and other 11 host genes. Therefore, pathogenic virulence genes could activate the expression of 11 host immune genes to facilitate all-round encirclement and suppression of pathogens, by stimulating the expression of *IL6* and then amplifying through signal transduction cascade. Notably, four host immune-related genes, *IL6, IL1R2, IL1B*, and *TLR5*, performed key regulatory roles in the interaction network. Moreover, *IL6* and *IL1R2* belonged to Group 4 of the host genes, so the expression of *htpG* could influence host immunity and thus activate the expression of *IL6* and *IL1R2*.
  In terms of the correlations between the virulence genes and host protein metabolism-related genes (Figure 6), firstly, it seems that when the infection occurs, the host immune and tissue cells could sense the presence of *P. plecoglossicida*, which will influence host immunity and then attenuate the expression of the pathogen virulence genes. Interestingly, the expression of host protein metabolism-related genes were also attenuated when infection happened, suggesting that *P. plecoglossicida* might cause white spot symptoms by disrupting host protein metabolism and damaging host spleen. Secondly, there were interactions between pathogenic genes and host genes. Among them, virulence genes such as *rpsS, rpsC, rplD, rplB, flgC, flgD, flgE, flgF*, and *flgG* could activated the expression of *CELA2*, indicating that *CELA2* might be an important host target for *P. plecoglossicida*. Moreover, there were mutual regulatory relationships between virulence genes. For example, *rplB* could promote the expression of *rplW, rplV, rplP, rpsH, rplR*, and *rpsE*; *rpsC* could promote the expression of *rplF, rpsK, rpsD*, and *rplQ*; and *rplD* could promote the expression of *rpoA*.
(2) Identification and visualization of hub virulence genes of *P. plecoglossicida*.
  A gene co-expression network of the virulence genes mentioned above was constructed and visualized (Figures 7, 8). Among the ribosomal genes, *rpsM, rpsJ, rplC, rplO, rpmD, rpsD, rpsE, rpoA, rpsC, rplR, rpsH, rplP, rpsK*, and *rplF* were the nodes of most connections in the network and considered as hub genes (Figure 7). Among flagellar genes, *flgD* was considered as a hub gene (Figure 8).
(3) Identification and visualization of hub response genes of *E. coioides*.
  A gene co-expression network of the host immune response-related genes and host protein metabolism-related genes mentioned above was constructed and visualized, respectively (Figures 9, 10). Among host immune response-related genes, *IL1B* was the node of most connections in the network and considered as a hub gene (Figure 9). Among host protein metabolism-related genes, *TRY, CPA1, CPA2*, and *CPB1* were considered as hub genes (Figure 10).

### Effects of the *htpG* Dependent Key Virulence Genes on the Pathogenicity of *P. plecoglossicida* in *E. coioides*

**Figure 11:**
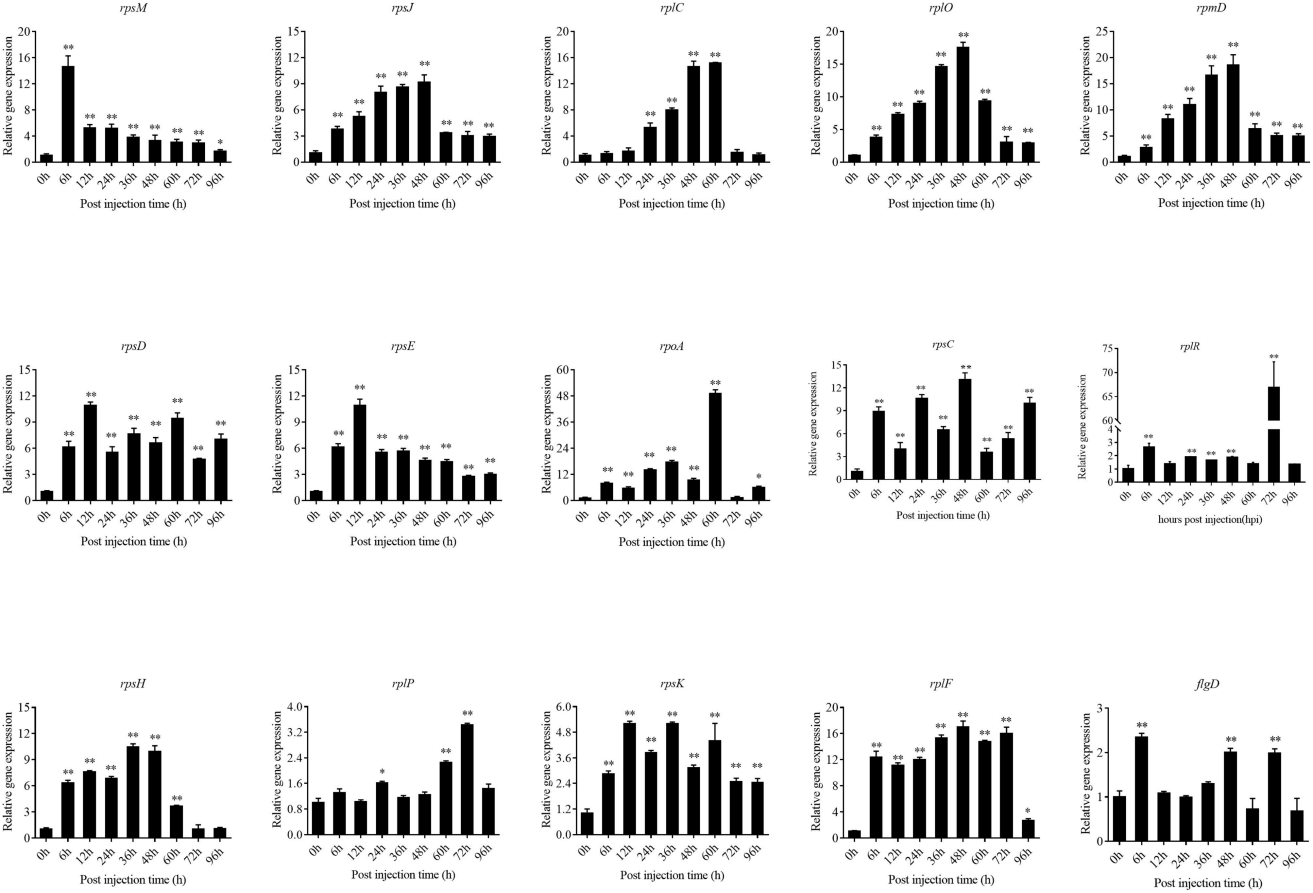
qRT-PCR analysis of the expression level of the predicted *htpG* dependent key genes during pathogen-host interactions in the wild type *P. plecoglossicida* infected spleen at different stages of infection. Data are presented as mean ± S.D. (*n* = 3). *P < 0.05, **P < 0.01.

**Figure 12:**
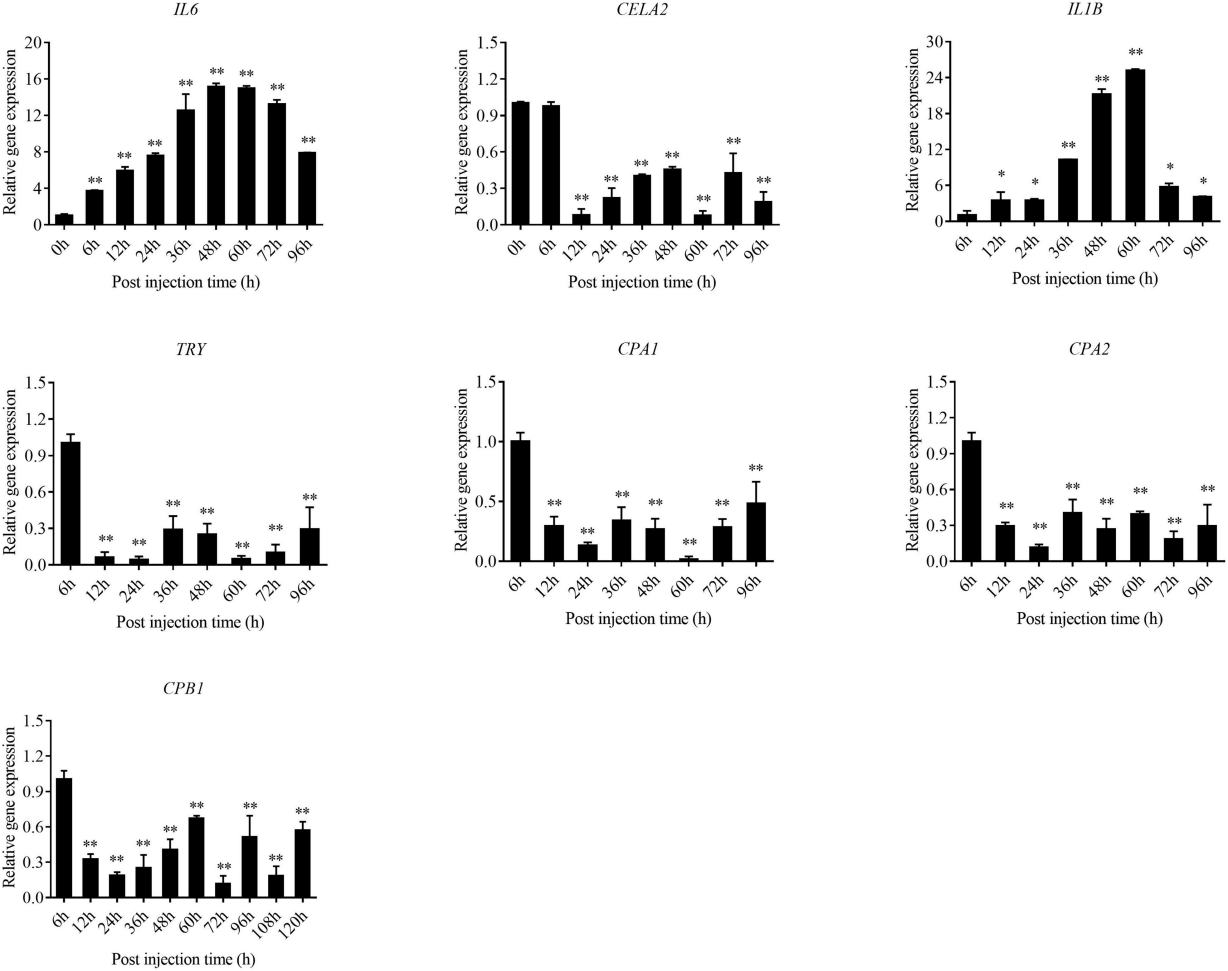
qRT-PCR analysis of the expression level of the hub response genes of *E. coioides* in the wild type *P. plecoglossicida* infected spleen at different stages of infection. Data are presented as mean ± S.D. (*n* = 3). *P < 0.05, **P < 0.01.

The expression level of the predicted *htpG* dependent key genes in pathogen-host interactions were evaluated in the wild-type *P. plecoglossicida* infected spleen at different stages of infection with qRT-PCR (Figure 11). The results showed that hub virulence genes of *P. plecoglossicida* were generally up-regulated throughout the infection process. For the hub response genes of *E. coioides, IL6* and *IL1B* were induced, while *CELA2, TRY, CPA1, CPA2*, and *CPB1* were repressed throughout the infection process (Figure 12). Meanwhile, the correlation tests showed that among the hub virulence genes of *P. plecoglossicida*, the ribosomal gene *rplF* was most significantly associated with *htpG* in the spleen during the infection (Table S3). Therefore, the ribosomal gene *rplF* and the flagellar gene *flgD* were chosen for further research.

**Figure 13:**
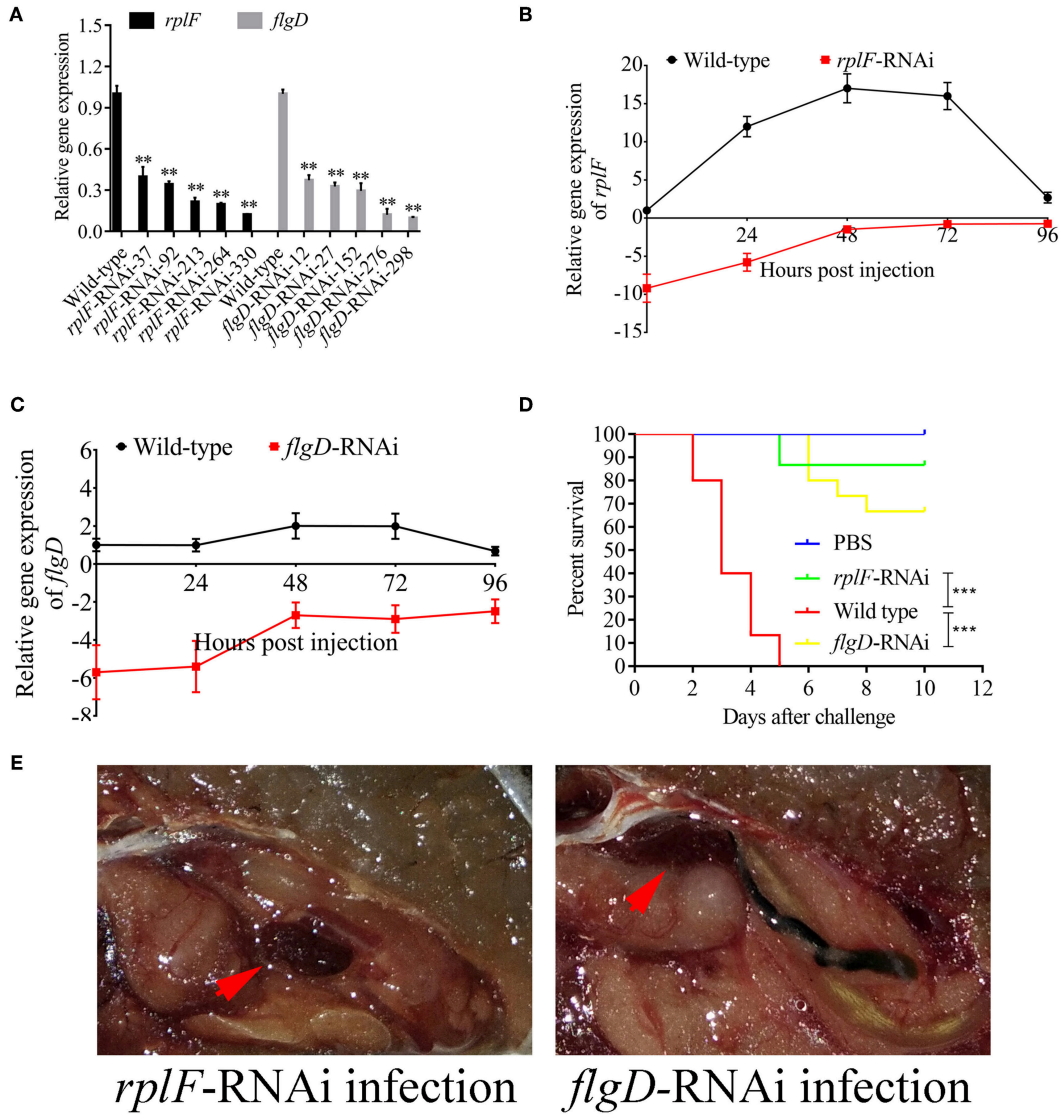
Construction of *rplF*- and *flgD*-RNAi strains and their effects on virulence. **(A)** Screening of shRNAs targeting *rplF* and *flgD* transcripts. Data are presented as mean ± S.D. (*n* = 3). **P < 0.01, ***p < 0.0001. **(B)** Expression level of *rplF* gene of *P. plecoglossicida* in the spleen of *E. coioides*. **(C)** Expression level of *rplF* gene of *P. plecoglossicida* in the spleen of *E. coioides*. **(D)** The cumulative survival of *E. coioides* injected with Wild type and RNAi strains during 10 days post challenge. **(E)** *E. coioides* that received *rplF*- or *flgD*-RNAi strains failed to develop visible nodules.

**Figure 14:**
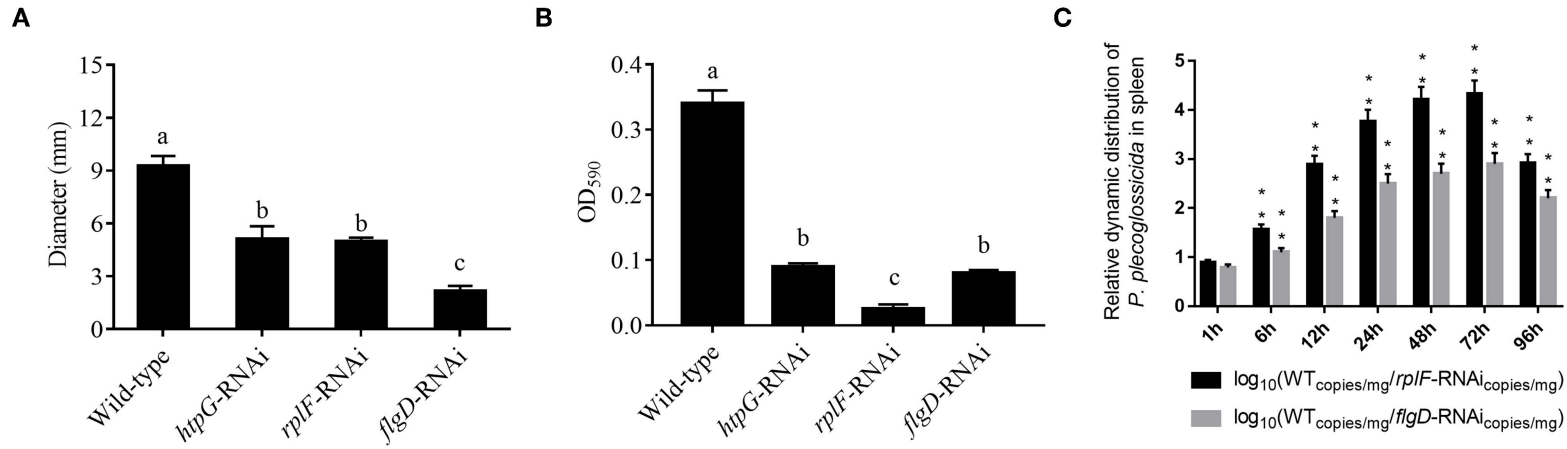
**(A)** Stable gene silencing reduces the motility behavior on soft agar plates of *P. plecoglossicida*. Diameter of the colony of each strain was presented as mean ± SD, *n* = 6. Means of treatments not sharing a common letter are significantly different at *P* < 0.05. **(B)** Biofilm formation of wild-type and stable silenced strains. Data are presented as mean ± S.D. (*n* = 6). Means of treatments not sharing a common letter are significantly different at *P* < 0.05. **(C)** The dynamic distribution of Wild type and RNAi strains in host spleen. Data are shown as means ± SD from three independent biological replicates. **P < 0.01.

*rplF* and *flgD* were stably silenced with different shRNAs resulting in different knockdown efficiencies (Figure 13A). The strains containing pCM130/tac-*rplF*-shRNA-330 (named the *rplF*-RNAi strain), and pCM130/tac-*flgD*-shRNA-298 (named the *flgD*-RNAi strain) showed the best efficiencies for silencing the *rplF* and *flgD* genes, respectively, and were used for further experiments. For 96 h post-infection, the expression of *rplF* (Figure 13B) and *flgD* (Figure 13C) in RNAi strains was always lower than wild-type strain in the spleen. The motility (Figure 14A) and biofilm formation (Figure 14B) of *P. plecoglossicida* was significantly decreased in the silenced strains. Meanwhile, the dynamic distributions of the *rplF*- and *flgD*-RNAi strains in *E. coioides* spleen were significantly reduced as compared to wild-type *P. plecoglossicida* at most of the time points after injection (Figure 14C). As compared to the wild-type strain, the mortality of *E. coioides* injected with RNAi strains decreased substantially (Figure 13D). Injection of RNAi strains also led to a delay in the time of death. Moreover, the spleens of all the *E. coioides* that received *rplF* or *flgD*-RNAi strains did not develop visible nodules (Figure 13E).

## Discussion

Dual RNA-seq was believed to be an ideal strategy to facilitate the understanding of “white nodules” disease, since it was capable of simultaneously monitoring host-pathogen transcriptome alterations during natural infections and offering a (46). However, the greatest obstacle to pathogen transcriptome sequencing in dual RNA-seq for pathogen-host interaction tissue samples is the extremely low abundance of pathogens (11). Therefore, simultaneous monitoring of the dynamics of gene expression changes between a bacterial pathogen and its host through tissue dual RNA-seq is currently lacking. The present study showed that *P. plecoglossicida* significantly accumulated in the spleen at 48 h, which was an ideal model for dual RNA-seq *in vivo* (Figure 2C). Therefore, we used a dual RNA-seq strategy to identify host-pathogen interaction regulators controlled by the expression of *htpG*.

The role of *htpG* remains highly elusive in bacterial pathogenicity recently, and the function of *htpG* during pathogen infection has not been reported (28). In the present study, *htpG* was found to be remarkably induced during the infection process. Meanwhile, silencing of *htpG* significantly reduced the distribution and virulence, but not the growth. Thus, *htpG* is a key activator of *P. plecoglossicida* virulence.

According to the results of dual RNA-seq, *htpG* was found to control the expression of 159 genes in *P. plecoglossicida*, directly or indirectly. Several pathways closely related to bacterial virulence regulation were involved, such as flagellar assembly, RNA degradation, ribosome, TCA cycle, glycolysis, and plant-pathogen interaction. Notably, distribution of DEGs positively related to *htpG* was classified, which finally achieved seven gene clusters covering 30 genes.

**Figure 15:**
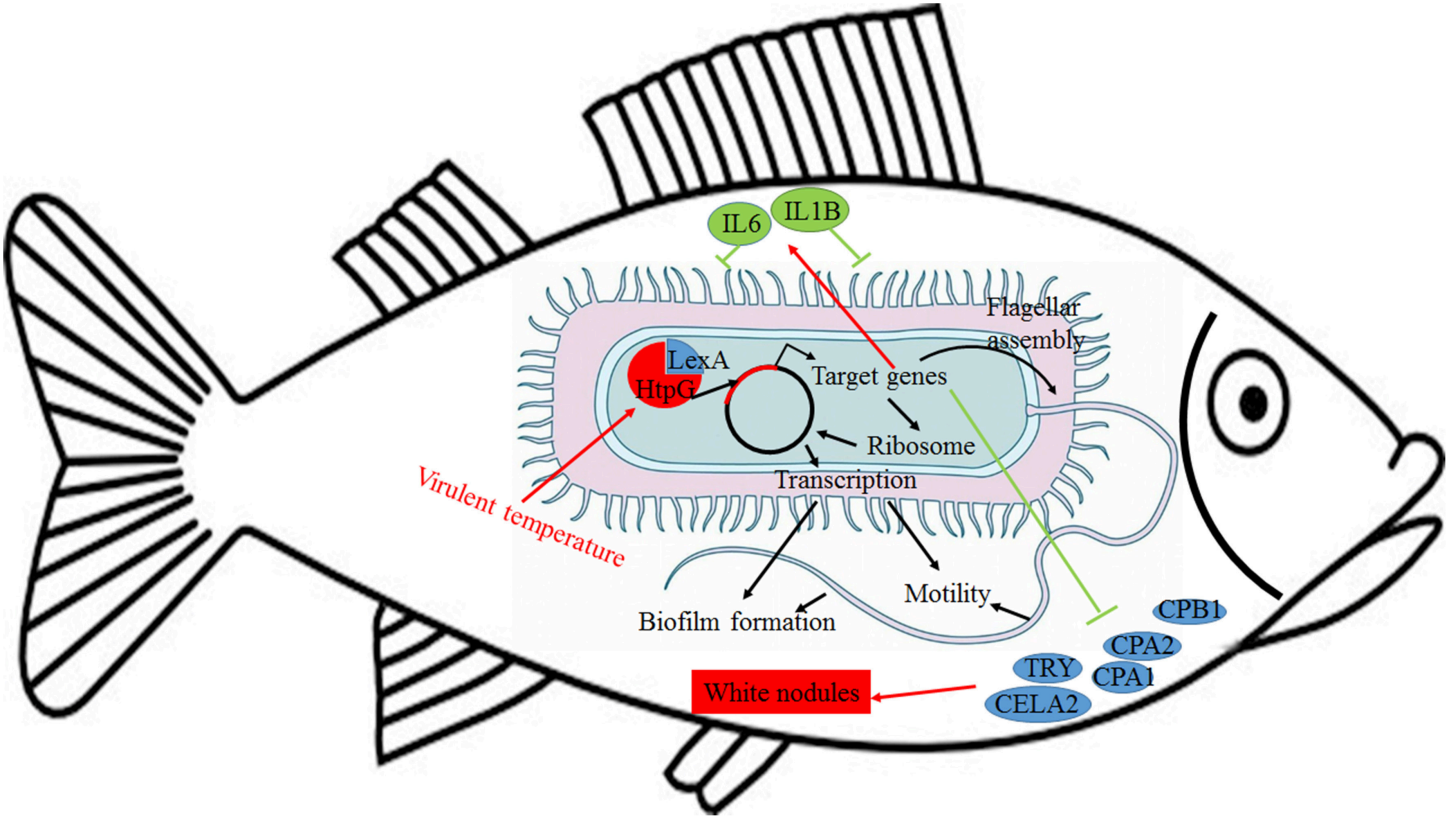
Schematic diagram to illustrate the hypothetical model of *P. plecoglossicida htpG* gene functions during host-pathogen interactions with *E. coioides*.

Further analysis of the seven gene clusters offered clues to how and why the *htpG*-dependent transcriptomes were generated. First, according to the RNA-seq data, the members in each gene cluster had similar expression profiles. Second, the members in each gene cluster were involved in a common pathway and performed similar functions. Third, distance analysis of the neighboring genes to these clusters indicated that most of the distances were within −10 to +50 base pairs, which are typical distances of operon structure genes in bacteria. These features led to a hypothesis that gene transcription in these seven clusters probably act as operons, whose activities are very sensitive to *htpG* expression level. HtpG has not yet been reported as a transcription factor. It acts as a chaperone that enables the optimal folding of newly synthesized proteins under stress conditions (22). Therefore, a hypothetical transcriptional regulator should exist. Transcription factor prediction analysis by BPROM showed that the seven clusters shared a common transcription factor LexA. Thus, HtpG probably ensured the optimal folding of LexA in adverse environment during infection, and then regulated the virulence-related genes in the seven clusters (Figure 15). Although further study is necessary to validate the hypothesis, *lexA* has been identified as a key gene of interest. Furthermore, as compared to the wild-type *P. plecoglossicida* infected spleen of *E. coioides*, numerous DEGs were identified from the *htpG-RNAi* strain infected spleen of *E. coioides*. Numerous pathways closely related to immune response regulation were identified, such as cytokine-cytokine receptor interaction, complement and coagulation cascades, lysosome, intestinal immune network for IgA production, systemic lupus erythematosus, primary immunodeficiency, NF-kappa B signaling pathway, antigen processing and presentation, chemokine signaling pathway, and Toll-like receptor signaling pathway (47, 48). This indicated that *htpG* gene played an important role in the immune response of *E. coioides* to *P. plecoglossicida*. In order to validate this, correlations between the virulence genes and host immune response-related genes were analyzed. Both *htpG*-independent (Group 3) and *htpG*-dependent (Group 4) defense mechanisms were observed. Infection activated the expression of immune-related genes and attenuated the expression of virulence genes. Meanwhile, there were interactions between pathogenic genes and host genes. Virulence genes such as *rplD, rplW, rplQ, flgD*, and *flgE* could influence host immunity and thus activate the expression of *IL6* expression, indicating that *IL6* played a key role in the interaction between pathogen and host. Finally, there were mutual regulatory relationships between host genes. Collectively, these observations strongly suggested that pathogenic virulence genes could activate the expression of 11 host immune genes to facilitate all-round encirclement and suppression of pathogens, by stimulating the expression of *IL6* and then amplifying through signal transduction cascade (Figure 15). Among the host immune response-related genes, *IL1B* was considered as a hub gene.

Interleukins (ILs) are the largest group of cytokines and play crucial roles in host innate/acquired immune response (49). To date, more than 30 ILs have been identified in fish (50). IL-6 as a key member of cytokines plays key roles in immune process, including acute phase response, hematopoiesis, energy sensor, and immune cell differentiation through modulating immune-signaling pathways (51, 52). Simultaneously, as a pro-inflammatory factor, up-regulation of IL-6 transcripts was detected in many fish defending against various pathogenic infections (53–55). Furthermore, IL-6 was found to drive lymphocyte differentiation after stimulation of trout head-kidney cells (56), indicating that it might be involved in fish immune response. Recent studies also revealed that IL-6 might be involved in large yellow croaker immune response and improve the inflammatory response through activating TNF-α expression. IL-1b, the first IL to be characterized, is crucial for the initiation and regulation of immune and inflammatory responses in many economically important teleost (57). Teleost IL-1b is also important for the host defense against pathogen infection (58). Recent studies suggested that LcIL-1β plays an important role in the large yellow croaker immune response against *V. alginolyticus* (57). Considering their crucial roles in fish inflammatory response, especially in the large yellow croaker, IL-6 and IL-1b may play important roles in the immune response of *E. coioides* to *P. plecoglossicida* during the process of white spot disease, and are closely related to the formation of granuloma. In the present study, *IL-6* and *IL-1B* were proven to be induced throughout the infection process by qRT-PCR, which reinforced the hypothesis.

Inflammation is associated with high levels of flagellin, the principal bacterial flagellar protein (59). Furthermore, TLR5 dependent innate-immunity-directed development of flagellin-specific adaptive immune responses can modulate the production of flagella in a three-way interaction that helps to avoid inflammation and tissue damage in mice. TLR5 could inhibit bacterial motility and down-regulate flagellar gene expression (60). In the present study, *TLR5* was proven to be a key immune-related gene and activated by *P. plecoglossicida* infection through dual RNA-seq. Meanwhile, flagellar assembly genes were predicted to be repressed during infection. Although the specific principles need to be further explored, these results indicated a three-way interaction dependent on TLR5 in *E. coioides*, which is closely related to the occurrence, development and regulation of inflammation during white spot disease.

The enzootic disease was characterized by granulomas in internal organs in cage-farmed large yellow croaker. The appearance of granulomas is usually caused by chronic inflammation in tissues (61). Previous histological analysis showed the accumulation of macrophages in the granulomatous lesions, which also indicated the presence of inflammation (62). However, the pathological cause of granulomas is unclear. Unexpectedly, we discovered correlations between the virulence genes and host protein metabolism-related genes. Interestingly, the expression of host protein metabolism-related genes were attenuated when infection occurred. Meanwhile, there were interactions between pathogenic genes and host genes. Among them, virulence genes such as *rpsS, rpsC, rplD, rplB, flgC, flgD, flgE, flgF*, and *flgG* could activate the expression of *CELA2*, indicating that *CELA2* might be an important target for *P. plecoglossicida* attacking to the host. Finally, there were mutual regulatory relationships among host protein metabolism-related genes. *TRY, CPA1, CPA2*, and *CPB1* were considered as hub genes and proven to be repressed throughout the infection process by qRT-PCR. Taken together, these results suggested that *P. plecoglossicida* might cause white spot symptoms by disrupting host protein metabolism and damaging the host spleen (Figure 15).

In the present study*, rplF* and *flgD* were predicted as *htpG*-dependent key virulence genes involved in host-pathogen interactions. Ribosomal protein L6, encoded by *rplF*, is essential for the assembly of functional 50S subunits at the late stage (63). FlgD, one of the flagellar scaffolding proteins, is required for flagellar hook assembly and acts as a hook-capping protein to enable assembly of hook protein subunits (64). Silencing of *htpG, rplF*, and *flgD* significantly reduced motility, biofilm formation, distribution and virulence. Taken together, these results indicated that *rplF* and *flgD* were *htpG*-dependent key virulence genes in *E. coioides*-*P. plecoglossicida* interactions. These results reflect the high sensitivity and accuracy of dual RNA-seq for predicting key virulence genes involved in host-pathogen interactions.

## Conclusion

This is the first report of the successful use of tissue dual RNA-seq to simultaneously monitor the dynamics of gene expression changes of pathogen and host in the context of prokaryotic and eukaryotic biological systems, which obtained high-resolution transcriptome data. Dual RNA-seq not only increased our knowledge of HtpG control of virulence gene expression in *P. plecoglossicida* during infection but also revealed the *htpG*-independent and *htpG*-dependent defense mechanisms of *E. coioides* against *P. plecoglossicida*. Furthermore, dual RNA-seq analysis also offered new clues underlying the pathological cause of granulomas. Notably, HtpG acts as a chaperone that enables the optimal folding of newly synthesized proteins under stress conditions, especially the change of temperature, while the virulence of *P. plecoglossicida* is temperature-dependent. Therefore, HtpG might act as a temperature sensor and effector, by regulating the expression of virulence genes, and then initiate the infection process at the pathogenic temperature. The ability of HtpG to respond to the environment is also likely to be activated by the adverse environment inside the host after the pathogen invasion, and then regulate the expression of virulence genes and interact with the host. In the future, we intend to validate these results and explain the molecular factors that govern these interactions during infection.

## Author Contributions

QY and YS conceived the experiments. LH, LZ, WL, XX, and YQ conducted the experiments. All authors assisted in the collection and interpretation of data. LH and QY wrote the manuscript.

### Conflict of Interest Statement

The authors declare that the research was conducted in the absence of any commercial or financial relationships that could be construed as a potential conflict of interest.

## Supplementary Material

Figure S1 Expression level of *htpG* of wild type *P. plecoglossicida* in the spleen of *E. coioides* during the infection. ^*^*p* < 0.05, ^***^*p* < 0.0001.

Figure S2 Hierarchical clustering of genes among wild type *P. plecoglossicida in vitro*, wild type *P. plecoglossicida in vivo*, and *htpG-RNAi* strain *in vivo*. For hierarchical clustering, green and red indicate decreased and increased expression, respectively. Transcripts were clustered by hierarchical clustering using the complete linkage algorithm and Pearson correlation metric in R.

Figure S3 K-means cluster analysis of 159 *P. plecoglossicida* genes from the *htpG-RNAi* strain in infected spleen of *E. coioides* exhibited significant difference.

Figure S4 Hierarchical clustering of genes among *E. coioides* accepted injection of PBS, wild type *P. plecoglossicida* and *htpG-RNAi* strain. For hierarchical clustering, green and red indicate decreased and increased expression, respectively. Transcripts were clustered by hierarchical clustering using the complete linkage algorithm and Pearson correlation metric in R.

Figure S5 K-means cluster analysis of 17512 *E. coioides* genes from the *htpG-RNAi* strain infected spleen of *E. coioides* exhibited significant difference.

Table S1 Oligonucleotides used in producing shRNA for stable gene silencing.

Table S2 Primers for qRT-PCR.

Table S3 Correlations between gene expressions.
