# Supplementary figures and images for: Dual RNA-Seq Unveils Pseudomonas plecoglossicida htpG Gene Functions During Host-Pathogen Interactions With Epinephelus coioides
Source: Front Immunol. 2019 May 3;10:984. doi: 10.3389/fimmu.2019.00984 (PMC6509204; doi:10.3389/fimmu.2019.00984)

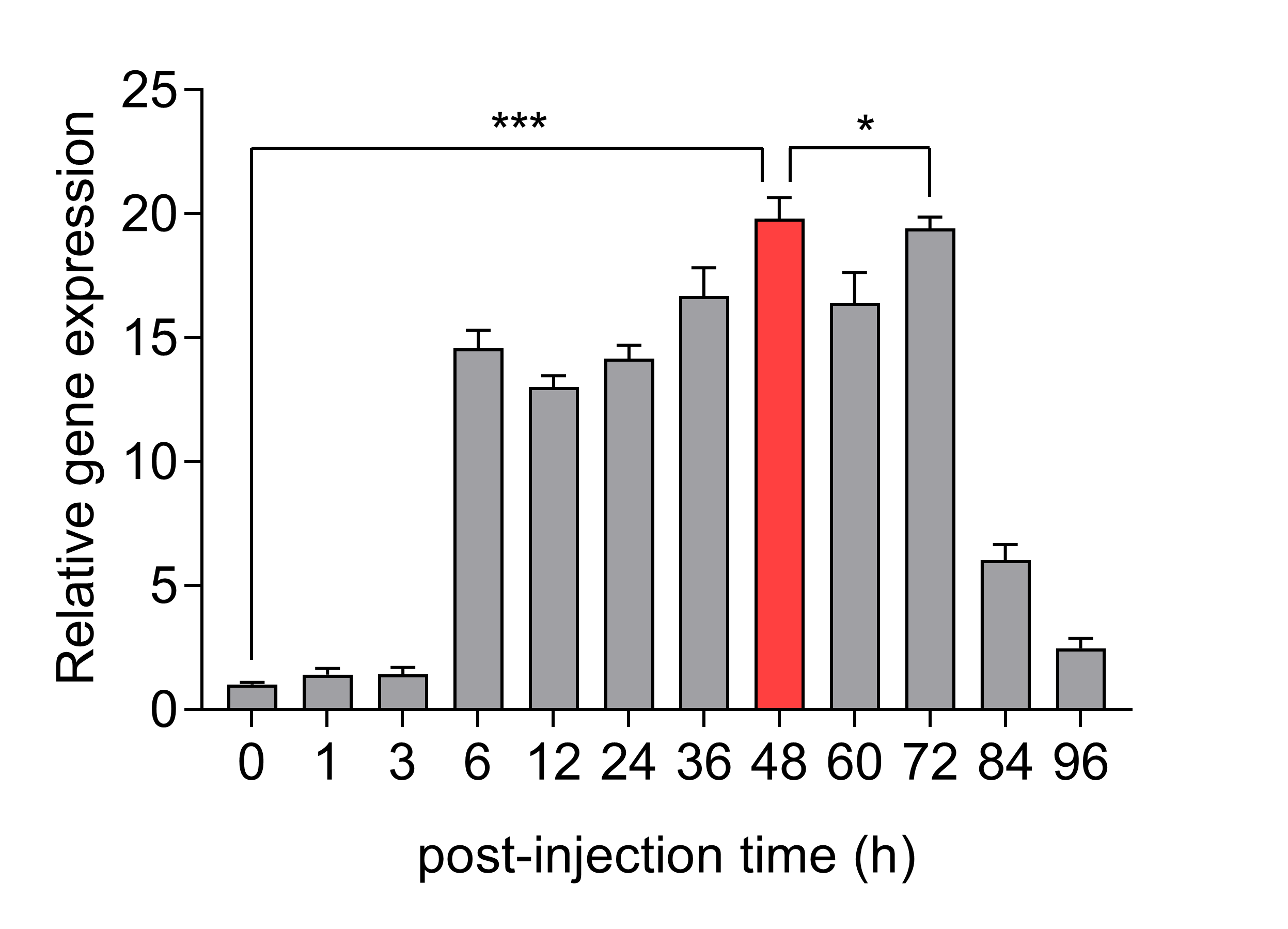

Supplement: Figure S1 — Expression level of htpG of wild type P. plecoglossicida in the spleen of E. coioides during the infection. *p < 0.05, ***p < 0.0001. [file Image_1.TIF]

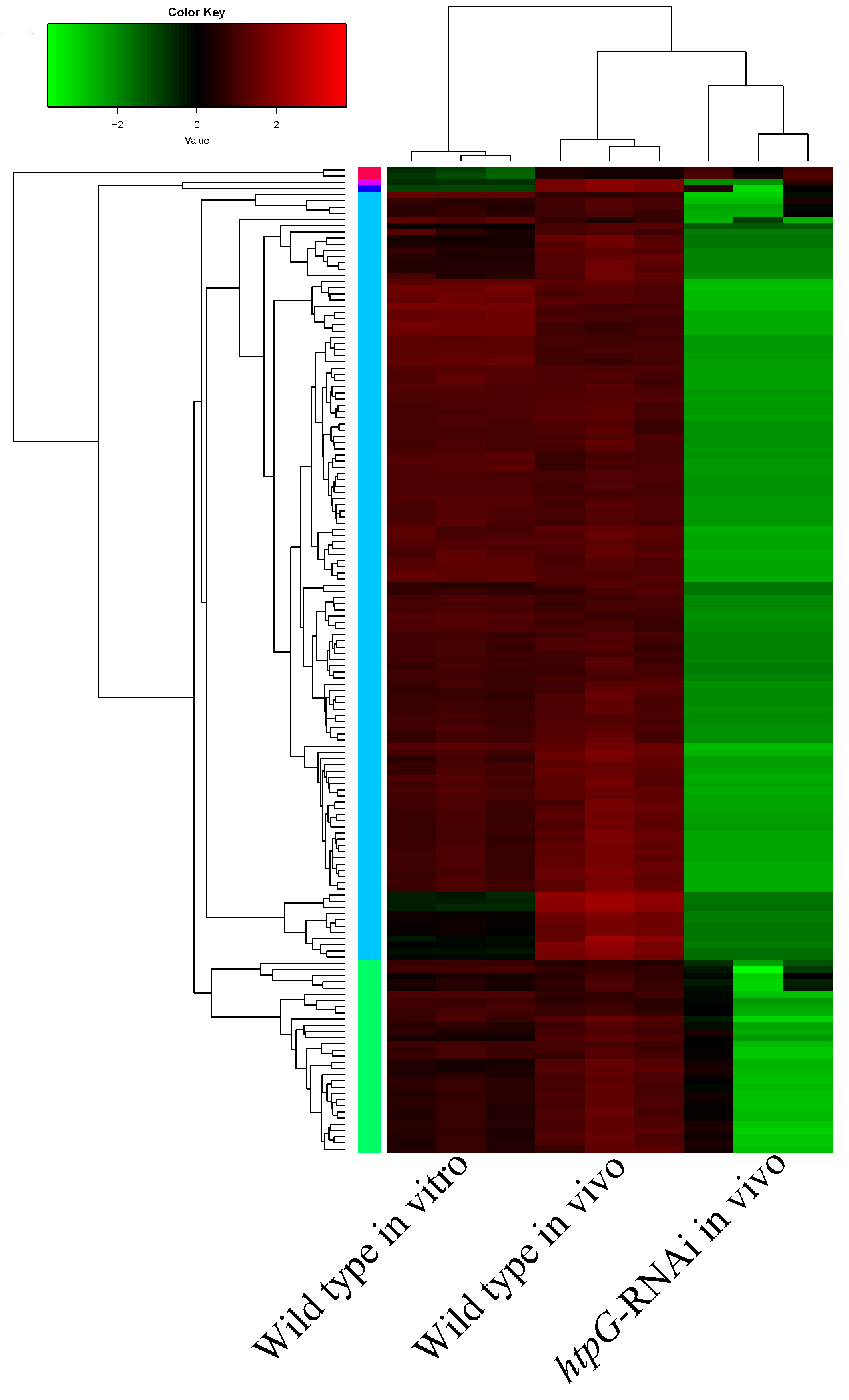

Supplement: Figure S2 — Hierarchical clustering of genes among wild type P. plecoglossicida in vitro, wild type P. plecoglossicida in vivo, and htpG-RNAi strain in vivo. For hierarchical clustering, green and red indicate decreased and increased expression, respectively. Transcripts were clustered by hierarchical clustering using the complete linkage algorithm and Pearson correlation metric in R. [file Image_2.TIF]

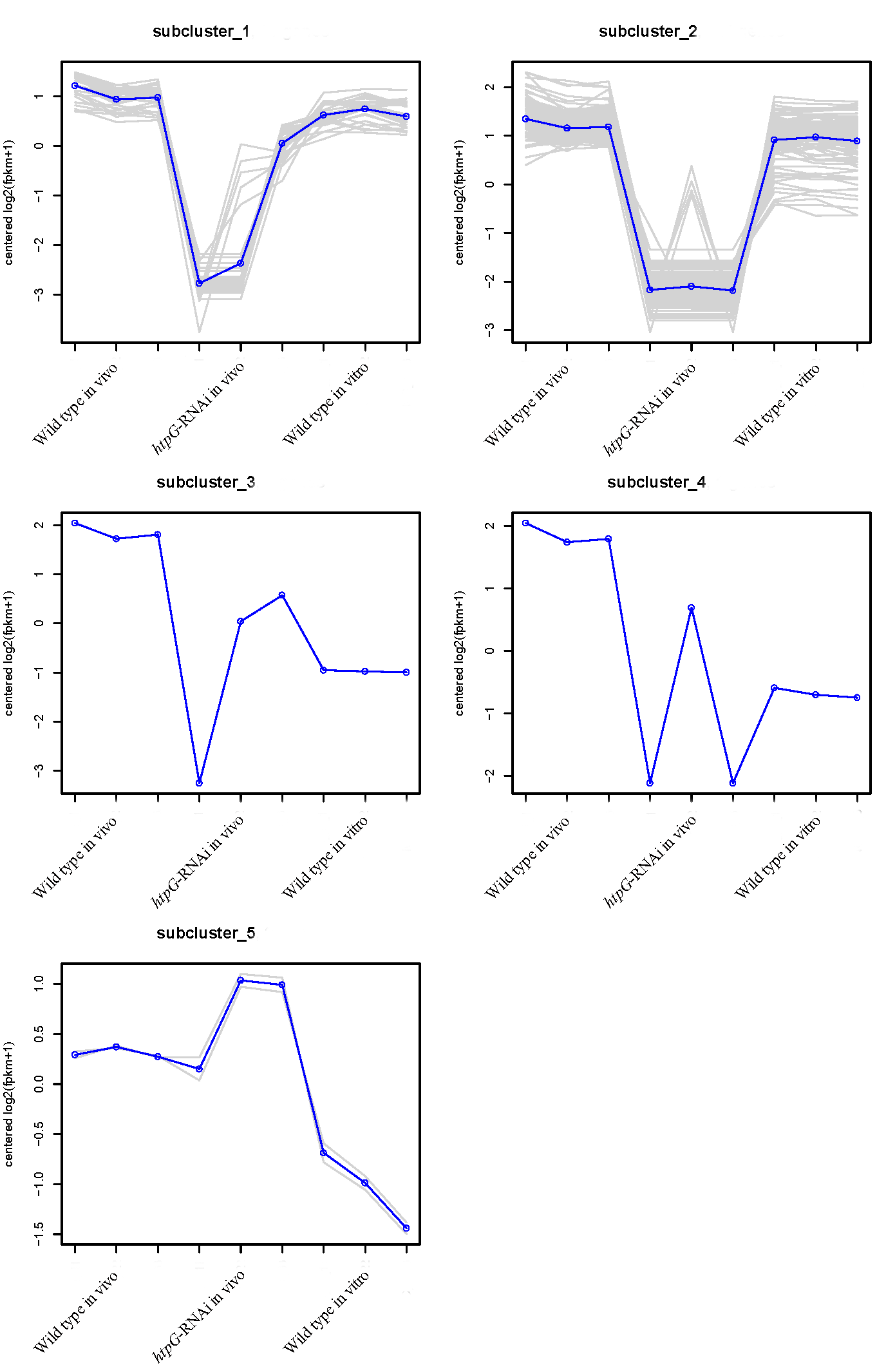

Supplement: Figure S3 — K-means cluster analysis of 159 P. plecoglossicida genes from the htpG-RNAi strain in infected spleen of E. coioides exhibited significant difference. [file Image_3.TIF]

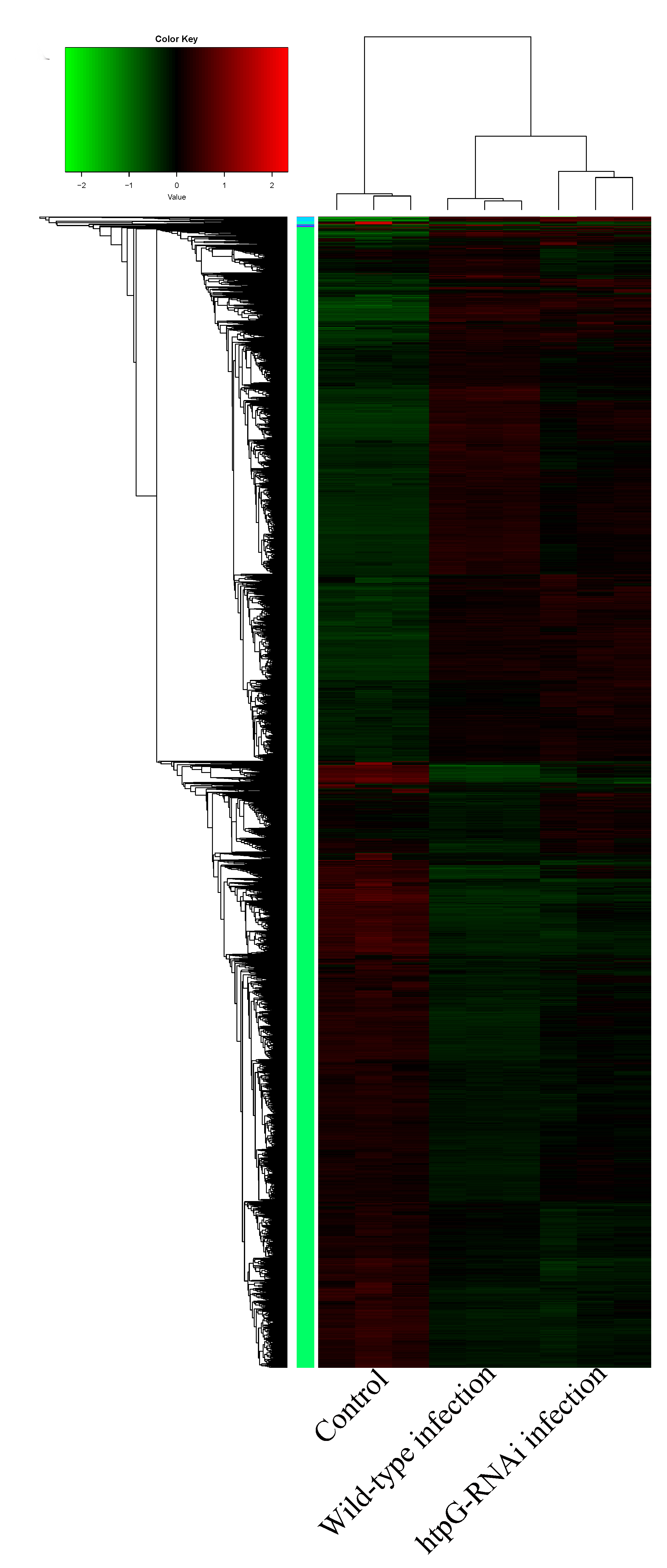

Supplement: Figure S4 — Hierarchical clustering of genes among E. coioides accepted injection of PBS, wild type P. plecoglossicida and htpG-RNAi strain. For hierarchical clustering, green and red indicate decreased and increased expression, respectively. Transcripts were clustered by hierarchical clustering using the complete linkage algorithm and Pearson correlation metric in R. [file Image_4.TIF]

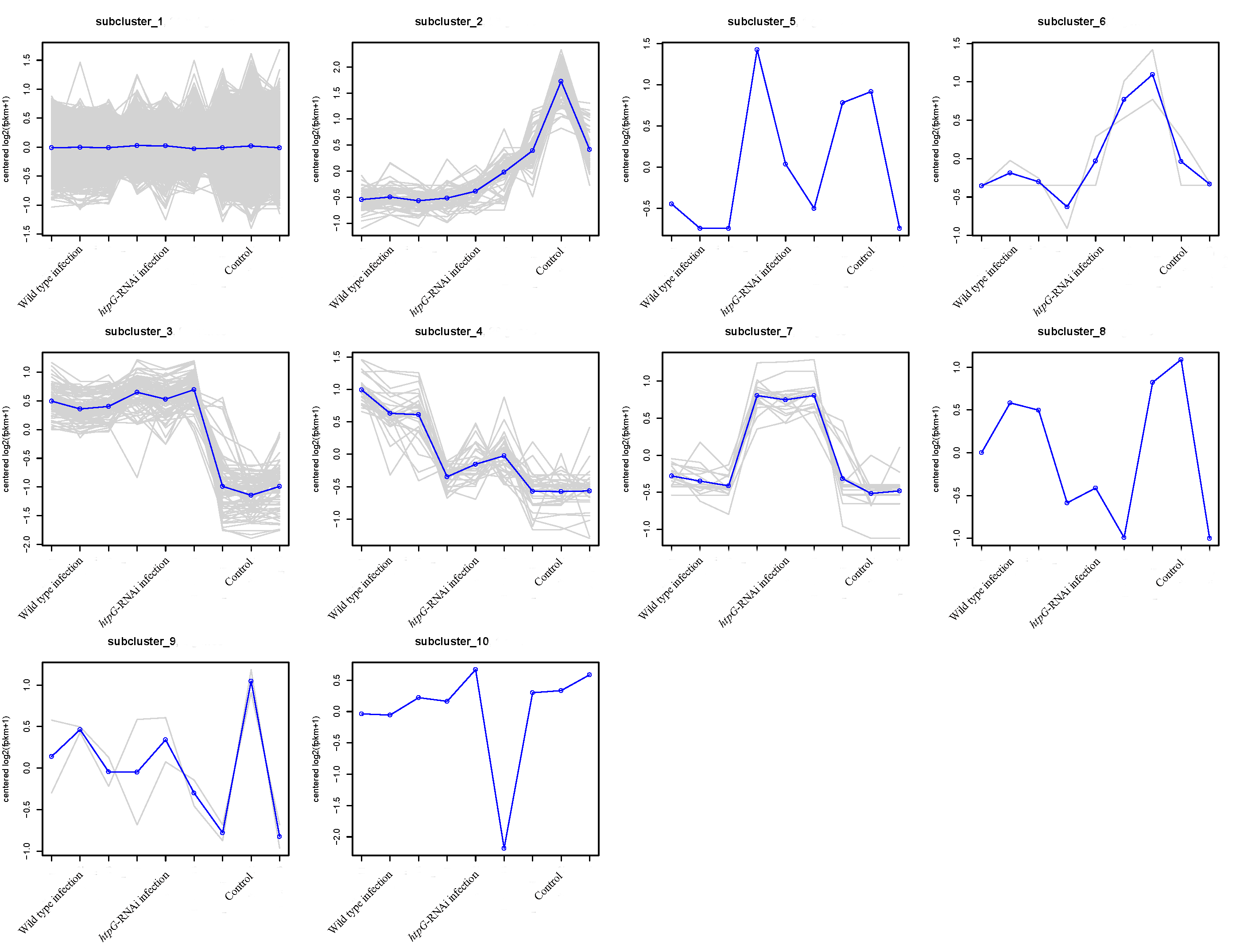

Supplement: Figure S5 — K-means cluster analysis of 17512 E. coioides genes from the htpG-RNAi strain infected spleen of E. coioides exhibited significant difference. [file Image_5.TIF]
