# Supplementary material for: Dual RNA-Seq Unveils Pseudomonas plecoglossicida htpG Gene Functions During Host-Pathogen Interactions With Epinephelus coioides
Source: Front Immunol. 2019 May 3;10:984. doi: 10.3389/fimmu.2019.00984 (PMC6509204; doi:10.3389/fimmu.2019.00984)
Supplement: Table S1 — Oligonucleotides used in producing shRNA for stable gene silencing. [file Table_1.DOC]

**Table S1** Oligonucleotides used in producing shRNA for stable gene silencing

| **Target gene** | **shRNA sequence for** **stable gene silence** |
| --- | --- |
| *htpG*-RNAi-57 | F:5'-TGCTGCACCTCATGATTCATTCTTCAAGAGAGAATGAATCATGAGGTGCAGCTTTTTTT-3'  R:5'-GTACAAAAAAAGCTGCACCTCATGATTCATTCTCTCTTGAAGAATGAATCATGAGGTGCAGCATGCA-3' |
| *htpG*-RNAi-340 | F:5'-TGGAGATCTTCCTTCGTGAACTTTCAAGAGAAGTTCACGAAGGAAGATCTCCTTTTTTT-3'  R:5'-GTACAAAAAAAGGAGATCTTCCTTCGTGAACTTCTCTTGAAAGTTCACGAAGGAAGATCTCCATGCA-3' |
| *htpG*-RNAi-603 | F:5'-TGGTGACCAGAAGAAGGATTCGTTCAAGAGACGAATCCTTCTTCTGGTCACCTTTTTTT-3'  R:5'-GTACAAAAAAAGGTGACCAGAAGAAGGATTCGTCTCTTGAACGAATCCTTCTTCTGGTCACCATGCA-3' |
| *htpG*-RNAi-819 | F:5'-TGGCAAGCTCGAATACAGTTCGTTCAAGAGACGAACTGTATTCGAGCTTGCCTTTTTTT-3'  R:5'-GTACAAAAAAAGGCAAGCTCGAATACAGTTCGTCTCTTGAACGAACTGTATTCGAGCTTGCCATGCA-3' |
| *htpG*-RNAi-826 | F:5'-TGGAAGTCTTCCGCAAGAAAGGTTCAAGAGACCTTTCTTGCGGAAGACTTCCTTTTTTT-3'  R:5'-GTACAAAAAAAGGAAGTCTTCCGCAAGAAAGGTCTCTTGAACCTTTCTTGCGGAAGACTTCCATGCA-3' |
| *rplF*-RNAi-37 | F:5'-TGCAGGCGTCGAAGTCAAATTCTTCAAGAGAGAATTTGACTTCGACGCCTGCTTTTTTT-3'  R:5'-GTACAAAAAAAGCAGGCGTCGAAGTCAAATTCTCTCTTGAAGAATTTGACTTCGACGCCTGCATGCA-3' |
| *rplF*-RNAi-92 | F:5'-TGCACTCTCGAACTGAACGTTCTTCAAGAGAGAACGTTCAGTTCGAGAGTGCTTTTTTT-3'  R:5'-GTACAAAAAAAGCACTCTCGAACTGAACGTTCTCTCTTGAAGAACGTTCAGTTCGAGAGTGCATGCA-3' |
| *rplF*-RNAi-213 | F:5'-TGGTCAACAACATGGTCCAAGGTTCAAGAGAGGTCAACAACATGGTCCAAGGTTTTTTT-3'  R:5'-GTACAAAAAAACCTTGGACCATGTTGTTGACCTCTCTTGAACCTTGGACCATGTTGTTGACCATGCA-3' |
| *rplF*-RNAi-264 | F:5'-TGTCAACAACATGGTCCAAGGCTTCAAGAGAGCCTTGGACCATGTTGTTGACTTTTTTT-3'  R:5'-GTACAAAAAAAGTCAACAACATGGTCCAAGGCTCTCTTGAAGCCTTGGACCATGTTGTTGACATGCA-3' |
| *rplF*-RNAi-330 | F:5'-TGCTGGTCGGTGTTGGTTACAATTCAAGAGATTGTAACCAACACCGACCAGCTTTTTTT-3'  R:5'-GTACAAAAAAAGCTGGTCGGTGTTGGTTACAATCTCTTGAATTGTAACCAACACCGACCAGCATGCA-3' |
| *flgD*-RNAi-12 | F:5'-TGCATAACGACACGTCGAACAATTCAAGAGATTGTTCGACGTGTCGTTATGCTTTTTTT-3'  R:5'-GTACAAAAAAAGCATAACGACACGTCGAACAATCTCTTGAATTGTTCGACGTGTCGTTATGCATGCA-3' |
| *flgD*-RNAi-27 | F:5'-TGAACAATGTCCAGGCCAATGATTCAAGAGATCATTGGCCTGGACATTGTTCTTTTTTT-3'  R:5'-GTACAAAAAAAGAACAATGTCCAGGCCAATGATCTCTTGAATCATTGGCCTGGACATTGTTCATGCA-3' |
| *flgD*-RNAi-152 | F:5'-TGCGAGTTTCTCAATCAGTTCGTTCAAGAGACGAACTGATTGAGAAACTCGCTTTTTTT-3'  R:5'-GTACAAAAAAAGCGAGTTTCTCAATCAGTTCGTCTCTTGAACGAACTGATTGAGAAACTCGCATGCA-3' |
| *flgD*-RNAi-276 | F:5'-TGGATAAGACCGTCAAGGTATGTTCAAGAGACATACCTTGACGGTCTTATCCTTTTTTT-3'  R:5'-GTACAAAAAAAGGATAAGACCGTCAAGGTATGTCTCTTGAACATACCTTGACGGTCTTATCCATGCA-3' |
| *flgD*-RNAi-298 | F:5'-TGATAAGACCGTCAAGGTATGCTTCAAGAGAGCATACCTTGACGGTCTTATCTTTTTTT-3'  R:5'-GTACAAAAAAAGATAAGACCGTCAAGGTATGCTCTCTTGAAGCATACCTTGACGGTCTTATCATGCA-3' |
