# Supplementary material for: Dual RNA-Seq Unveils Pseudomonas plecoglossicida htpG Gene Functions During Host-Pathogen Interactions With Epinephelus coioides
Source: Front Immunol. 2019 May 3;10:984. doi: 10.3389/fimmu.2019.00984 (PMC6509204; doi:10.3389/fimmu.2019.00984)
Supplement: Table S2 — Primers for qRT-PCR. [file Table_2.DOC]

**Table S2. Primers for qRT-PCR**

| **Gene** | **Primers** |
| --- | --- |
| *c129279_g3* | F:5'-GCCGAAGAAGGTCGAAGTGG-3'  R:5'-GTTGTGAACCCAACTGGT-3' |
| *C2* | F:5'-TGGAGAAAGCGTCGTATC-3'  R:5'-CTGTTCTGGCTGAAGGAG-3' |
| *c321660_g6* | F:5'-TTGAGCGTATGGACTTCCC-3'  R:5'-CGGTTTCTTCGTCGGTCTT-3' |
| *c47659_g2* | F:5'-GACAGGGCTGCCTTATTG-3'  R:5'-GCAGAACTTCTTCAGGTGGT-3' |
| *C7* | F:5'-ATGATGGACAGGGC AGAG-3'  R:5'-GAGCCACATCGTAGTCGTAA-3' |
| *CELA2* | F:5'-GTCTCTGCAGTACAAGAGTG-3'  R:5'-CCTCCAGCACAGACCATGTT-3' |
| *clpA* | F:5'-CCCGTCGTCGCAAGAACA-3'  R:5'-CCAGCAGCGCACCAAGAT-3' |
| *CPA1* | F:CATAGCGACCAGTGTCCCTCA-3'  R:CGGCTACTCCTGCAAGAACG-3' |
| *CPA2* | F:ATTGTCGGAAACAGTATGGTGG-3'  R:GGAAGAAGATGCTGGTGTAGAGG-3' |
| *CPB1* | F:CGGCTAGACTGCTGTTGGGT-3'  R:GGCTTTGGATGTTATCATATTGGTA-3' |
| *dnaK* | F:5'-ACGGCGAAATCCTGGTAG-3'  R:5'-CATTGCTTGCCTTGACGA-3' |
| *flgB* | F:5'-CAGCGTAGCGAAATCCTTGC-3'  R:5'-TGCCGGTGACGACTGGTAT-3' |
| *flgD* | F:5'-GCGCCGAAATGCCATTAC-3'  R:5'-TGAATCCGCTGATACGACTGA-3' |
| *fliG* | F:5'-GTTGGGCAGCGACATTACC-3'  R:5'-CAAGAAAGCCAGGAACACG-3' |
| *fusA* | F:5'-TTGAGCGTATGGACTTCCC-3'  R:5'-CGGTTTCTTCGTCGGTCTT-3' |
| *htpG* | F:5'-GAAGAACGAGGCGGAGCA-3'  R:5'-CGGTCGGTCAGCAGCAATA-3' |
| *IL1B* | F:5'-AGCCACAGTTCTGCCCTCAA-3'  R:5'-CCAACCTCATCATCGCCAC-3' |
| *IL6* | F:5'-TCCCGACACTCCGCTATCTT-3'  R:5'-TGGCGAGTTTCCACTGATGC-3' |
| *pldA* | F:5'-CCTTCCTCCAACTTCAGC-3'  R:5'-ATCAGGGTTCCTCATCTTTC-3' |
| *rplC* | F:5'-GCAAGATCAGCCAGTACACC-3'  R:5'-CAACGACATAGAAGCCAAGAC-3' |
| *rplE* | F:5'-GTCATCGAGCACGCTGTT-3'  R:5'-CCATCCTTCACGGACTTTG-3' |
| *rplF* | F:5'-TGTTGGTTACAAGGCACAGG-3'  R:5'-TGTCGGTCTGGCTAGGAGT-3' |
| *rplN* | F:5'-CCTTGACGGTAACCTTGATG-3'  R:5'-TCGATGTGGCCGATAACA-3' |
| *rplO* | F:5'-AAGTTCGGCTTCGTTTCCC-3'  R:5'-CGCTGAATGTGCTGGTTGAT-3' |
| *rplP* | F:5'-ATTCCGCAAGCAGATGACC-3'  R:5'-TTACCTTTACCCATACGAACCTC-3' |
| *rplR* | F:5'-GCACATCTACGCCCAGGTCA-3'  R:5'-CGCCTTCACGAGCAGCAT-3' |
| *rplW* | F:5'-TGGCTGAAGGGAGACGAT-3'  R:5'-CAAGGTTGCTACCGATGC-3' |
| *rpmD* | F:5'-GCTGAAGAAGGTCGAAGTGG-3'  R:5'-GTTGTGAACCGCGATGGT-3' |
| *rpoA* | F:5'-GACGAAAGCCGTAGCATTGG-3'  R:5'-GCTCGCTGTCACCTTTGAGG-3' |
| *rpsC* | F:5'-CGAGAAGCTGCGTCAGGA-3'  R:5'-ACGACCGCTCACCTGGAT-3' |
| *rpsD* | F:5'-CGTATCTACGGTGTTCTGGAGCG-3'  R:5'-ACCCGGACGAACTTGGTAGGAT-3' |
| *rpsE* | F:5'-GCGACGAAGGCTACATCGAG-3'  R:5'-CTGGATCATGTTGCGACGAG-3' |
| *rpsH* | F:5'-CGGTTGCCAAAGTTCTGAAAG-3'  R:5'-TGTTGGTGGAGACGATAGACAC-3' |
| *rpsJ* | F:5'-GTCATCGAGCACGCTGTT-3'  R:5'-CCATCCTTCACGGACTTTG-3' |
| *rpsK* | F:5'-TTTGTCCTGGGCGACCTC-3'  R:5'-ACCCGTTGTGCGGGATT-3' |
| *rpsM* | F:5'-TGTTGGTTACAAGGCACAGG-3'  R:5'-TGTCGGTCTGGCTAGGAGT-3' |
| *rpsS* | F:5'-GCTGAAGAAGGTCGAAGTGG-3'  R:5'-GTTGTGAACCGCGATGGT-3' |
| *secY* | F:5'-GCAAGATCAGCCAGTACACC-3'  R:5'-CAACGACATAGAAGCCAAGAC-3' |
| *TLR5* | F:5'-TCACATTGGTGTTCTGGGTC-3'  R:5'-CGGCGAGTCTATCAGTTTAT-3' |
| *TRY* | F:5'-GGTACAGGAGCATCTGGGAGTA-3'  R:5'-AGCAACCGTATGTGGCGTAA-3' |
| *tuf* | F:5'-GCCTACATCCCTGAGCCA-3'  R:5'-GGTATCACGCAGACCAACG-3' |
| *gyrB* | F:5'-TGCTGAAGGACGAGCGTTCG-3'  R:5'-ATCATCTTGCCGACAACAGC-3' |
| *16S RNA* | F:5'-GTTGGGAGGAAGGGCAGTAAG-3'  R:5'-ATCTAGGCATTTCACCGCTACA-3' |
| *β-actin* | F:5'-ACGCTCTGCCTCACGCCATC-3'  R:5'-GGTGGTGAAGCTGTAGCC-3' |
