# Supplementary material for: Dual RNA-Seq Unveils Pseudomonas plecoglossicida htpG Gene Functions During Host-Pathogen Interactions With Epinephelus coioides
Source: Front Immunol. 2019 May 3;10:984. doi: 10.3389/fimmu.2019.00984 (PMC6509204; doi:10.3389/fimmu.2019.00984)
Supplement: Table S3 — Correlations between gene expressions. [file Table_3.DOCX]

**Table S3. Correlations between gene expressions**

|  | **Pearson r** | **P value** |
| --- | --- | --- |
| *htpG* vs *rplF* | 0.9956 | <0.0001 |
| *htpG* vs *rpsM* | 0.3023 | 0.4292 |
| *htpG* vs *rpsJ* | 0.5996 | 0.0879 |
| *htpG* vs *rplC* | 0.5330 | 0.1395 |
| *htpG* vs *rplO* | 0.6271 | 0.0707 |
| *htpG* vs *rpmD* | 0.5714 | 0.1080 |
| *htpG* vs *rpsD* | 0.4489 | 0.2255 |
| *htpG* vs *rpsE* | 0.3993 | 0.2871 |
| *htpG* vs *rpoA* | 0.3417 | 0.3681 |
| *htpG* vs *rpsC* | 0.2944 | 0.4420 |
| *htpG* vs *rplR* | 0.3379 | 0.3738 |
| *htpG* vs *rpsH* | 0.5776 | 0.1034 |
| *htpG* vs *rplP* | 0.4240 | 0.2554 |
| *htpG* vs *rpsK* | 0.5818 | 0.1003 |
